# CAPTURE: a multinational, cross-sectional study of cardiovascular disease prevalence in adults with type 2 diabetes across 13 countries

**DOI:** 10.1186/s12933-021-01344-0

**Published:** 2021-07-27

**Authors:** Ofri Mosenzon, Abdullah Alguwaihes, Jose Luis Arenas Leon, Fahri Bayram, Patrice Darmon, Timothy M. E. Davis, Guillermo Dieuzeide, Kirsten T. Eriksen, Tianpei Hong, Margit S. Kaltoft, Csaba Lengyel, Nicolai A. Rhee, Giuseppina T. Russo, Shinichiro Shirabe, Katerina Urbancova, Sergio Vencio, the CAPTURE Study Investigators

**Affiliations:** 1 Diabetes Unit, Department of Endocrinology and Metabolism, Hadassah Medical Center, Faculty of Medicine, Hebrew University of Jerusalem, Hadassah Hebrew University Hospital, PO Box 12000, 91120 Jerusalem, Israel; 2 King Saud University, King Saud University Medical City, Riyadh, Kingdom of Saudi Arabia; 3 Centro de Atención E Investigación Cardiovascular del Potosí, San Luis Potosí, Mexico; 4 Department of Endocrinology and Metabolism, Faculty of Medicine, Erciyes University, Kayseri, Turkey; 5 Aix Marseille University, INSERM, INRA, C2VN, Marseille, France; 6 Medical School, University of Western Australia, Fremantle Hospital, Fremantle, Australia; 7 Centro de Atención Integral en Diabetes, Endocrinología Y Metabolismo, Chacabuco, Buenos Aires, Argentina; 8 Novo Nordisk A/S, Søborg, Denmark; 9 Department of Endocrinology and Metabolism, Peking University Third Hospital, Beijing, China; 10 University of Szeged, Szeged, Hungary; 11 Novo Nordisk Health Care AG, Zurich, Switzerland; 12 Department of Clinical and Experimental Medicine, Policlinico Universitario, University of Messina, Messina, Italy; 13 H. E. C Science Clinic, Yokohama, Japan; 14 Diabetologická Interní Ambulance S.R.O., Ostrava, Czech Republic; 15 Instituto de Ciencias Farmaceuticas, Goiânia, Goiás Brazil

**Keywords:** Non-interventional study, Type 2 diabetes, Cardiovascular disease, Atherosclerotic cardiovascular disease, Prevalence, Glucagon-like peptide-1 receptor agonists, Sodium-glucose co-transporter-2 inhibitors

## Abstract

**Background:**

There is a paucity of global data on cardiovascular disease (CVD) prevalence in people with type 2 diabetes (T2D). The primary objective of the CAPTURE study was to estimate the prevalence of established CVD and its management in adults with T2D across 13 countries from five continents. Additional objectives were to further characterize the study sample regarding demographics, clinical parameters and medication usage, with particular reference to blood glucose-lowering agents (GLAs: glucagon-like peptide-1 receptor agonists and sodium-glucose co-transporter-2 inhibitors) with demonstrated cardiovascular benefit in randomized intervention trials.

**Methods:**

Data were collected from adults with T2D managed in primary or specialist care in Australia, China, Japan, Czech Republic, France, Hungary, Italy, Argentina, Brazil, Mexico, Israel, Kingdom of Saudi Arabia, and Turkey in 2019, using standardized methodology. CVD prevalence, weighted by diabetes prevalence in each country, was estimated for the overall CAPTURE sample and participating countries. Country-specific odds ratios for CVD prevalence were further adjusted for relevant demographic and clinical parameters.

**Results:**

The overall CAPTURE sample included 9823 adults with T2D (n = 4502 from primary care; n = 5321 from specialist care). The overall CAPTURE sample had median (interquartile range) diabetes duration 10.7 years (5.6–17.9 years) and glycated hemoglobin 7.3% (6.6–8.4%) [56 mmol/mol (49–68 mmol/mol)]. Overall weighted CVD and atherosclerotic CVD prevalence estimates were 34.8% (95% confidence interval [CI] 32.7–36.8) and 31.8% (95% CI 29.7–33.8%), respectively. Age, gender, and clinical parameters accounted for some of the between-country variation in CVD prevalence. GLAs with demonstrated cardiovascular benefit were used by 21.9% of participants, which was similar in participants with and without CVD: 21.5% and 22.2%, respectively.

**Conclusions:**

In 2019, approximately one in three adults with T2D in CAPTURE had diagnosed CVD. The low use of GLAs with demonstrated cardiovascular benefit even in participants with established CVD suggested that most were not managed according to contemporary diabetes and cardiology guidelines.

*Study registration* NCT03786406 (registered on December 20, 2018), NCT03811288 (registered on January 18, 2019).

**Supplementary Information:**

The online version contains supplementary material available at 10.1186/s12933-021-01344-0.

## Introduction

Cardiovascular disease (CVD) remains the largest cause of diabetes-related morbidity and mortality [1, 2]. Systematic reviews indicate there is an approximately two-fold higher risk of CVD in people with versus without diabetes after adjustment for conventional risk factors [3, 4]. Given the large clinical burden associated with CVD complicating type 2 diabetes (T2D), international diabetes and cardiology position statements and guidelines have been updated to encompass the combined management of T2D and CVD [5–7]. These updates have been informed by cardiovascular (CV) outcome trials demonstrating superiority in CV outcomes for some blood glucose-lowering agents (GLAs) versus placebo in people with T2D and established CVD or at high CVD risk [6, 8]. The glucagon-like peptide-1 receptor agonists (GLP-1 RAs) and sodium-glucose co-transporter-2 inhibitors (SGLT2is) with demonstrated CV benefit are now recommended as first [5] or second-line [6, 7] GLAs in this context.

The impact of the updated guidelines on real-world clinical practice should be of interest to clinicians and policy makers, and its evaluation requires robust contemporary estimates of CVD prevalence and management in people with T2D that can be monitored over time. However, the current understanding of the global prevalence of, and between-country variation in, diabetes-related complications, including CVD, is limited [9]. Available CVD prevalence data in people with T2D are mostly from regional or national studies conducted in the US [10] and some European countries [11–15]. Direct comparisons between countries are complicated by differences in study methodology, including the population sampled and definition of CVD. Estimates of the CVD burden in some countries have been based on studies that sampled selected patient populations (such as from specialist care) or that utilized model-based estimates rather than real-world data [16]. A review paper estimated CVD to affect 32.2% of individuals with T2D worldwide, based on a systematic review of scientific literature published between 2007 and 2017 [16] that would not reflect recent changes in epidemiology and management of T2D and is now considered outdated.

The primary objective of the present study was to estimate the contemporary prevalence of established CVD in adults with T2D across a range of countries and several continents using standardized methodology. Secondary objectives were to estimate the prevalence of high CVD risk in adults with T2D without established CVD (full results to be published elsewhere) and to further characterize the study sample regarding demographics, clinical parameters, CV medication and GLA usage, with particular reference to GLAs (GLP-1 RAs and SGLT2is) with demonstrated CV benefit.

## Methods

### Study design

CAPTURE was a non-interventional, cross-sectional study conducted at 214 centers across 13 countries (NCT03786406 [Europe] and NCT03811288 [non-Europe]). The countries were from Australasia (Australia), East Asia (China and Japan), Europe (Czech Republic, France, Hungary, and Italy), Latin America (Argentina, Brazil, and Mexico), and the Middle East (Israel, Kingdom of Saudi Arabia and Turkey). The study protocols were approved by the appropriate independent ethics committees and relevant institutional review boards. The study was conducted in accordance with the Declaration of Helsinki [17], International Society for Pharmacoepidemiology Good Pharmacoepidemiology Practices [18], and local regulations. All participants provided written informed consent prior to participation.

Local medical affairs personnel from participating countries, who were employed by the sponsor, provided information on the management of people with T2D, including types of physicians managing T2D in routine clinical practice (primary care practitioners, diabetologists, endocrinologists, cardiologists, and other specialists) and types of practices (primary care and specialist settings, including different types of hospitals) to a contract research organization for consideration of site selection. Final participating sites were chosen by the contract research organization and approved by the sponsor on the basis that they were as representative as possible for each country. Factors considered were geographical spread and the division of patients being treated at private and public centers, as well as the degree of specialization at each site. The ratio of participants from primary to specialist care sites in each country was chosen to approximate the assumed distribution of adults with T2D managed in these settings according to available local data. No primary care sites were included in China, Hungary, or Italy as people with T2D in these countries are primarily, although not exclusively, managed in specialist care. In Italy, local health system organization rules mean that new prescriptions for GLAs cannot be provided in primary care. In China, patients with T2D consult hospital doctors as their primary contact. In Hungary, patients with T2D are mainly managed in a specialist care setting with limited influence from primary care on decisions regarding GLAs with CV benefit.

At each participating site, consecutive, eligible adults aged ≥ 18 years (≥ 20 years in Japan) at the time of informed consent who had been diagnosed with T2D ≥ 180 days prior to providing informed consent and who were attending the site as part of their routine visit to their treating physician were invited to participate by the treating physician during a 90-day time window. Exclusion criteria were a diagnosis of type 1 diabetes, known congenital heart disease or malformation, previous participation in this study (provided informed consent for inclusion at a prior visit during the data collection period), and mental incapacity or language barriers that precluded an adequate understanding of, or cooperation with, study requirements.

The invitation to participate, provision of informed consent, and data collection took place during a single, routine health visit at each site. In all countries, the treating physician or a trained delegate collected data using a standardized electronic case report form, and data were transferred to a central database via a web-based data capture system. Relevant data were collected from participants’ medical records. The physician verbally asked participants for any information that was missing from the medical record. The study protocols did not mandate screening for, or adjudication of, the presence of complications .

### Definitions of variables studied

Established CVD was defined as a diagnosis of any of the following conditions in participants’ medical records: cerebrovascular disease, coronary heart disease (CHD), heart failure, cardiac arrhythmia or conduction abnormalities, aortic disease, peripheral artery disease (PAD), or carotid artery disease (see Additional file 1: Table S1 for the full list and definitions). Similarly, CVD was categorized as atherosclerotic CVD (ASCVD) when there was a diagnosis of any of the following CV conditions: cerebrovascular disease, CHD, PAD, or carotid artery disease [7]. For analysis purposes, participants were stratified into two groups based on the presence (CVD group) or absence (non-CVD group) of established CVD.

Available demographic, anthropometric, and clinical parameters were collected, in addition to selected medical history, GLAs, and CV medications (listed in Additional file 1: Methods S1). Only current medications or those discontinued within the previous 3 months were recorded. During analysis, GLAs were further grouped according to demonstrated CV benefit status in line with the 2020 American Diabetes Association guidelines [19] (and by March 2020, all had a CV indication in their US Food and Drug Administration label [20–25]). GLAs with demonstrated CV benefit included three GLP-1 RAs (dulaglutide, liraglutide, and semaglutide) and three SGLT2is (canagliflozin, dapagliflozin, and empagliflozin).

### Statistical analysis

#### Pre-specified analyses

The prevalence (95% confidence interval [CI]) of CVD, ASCVD, CVD subtypes, and diagnoses were estimated for all countries together (overall) and for each country individually. Overall prevalence estimates were calculated as weighted estimates to account for the size of the diabetes population in each country [26], as this was not accounted for in the sampling. Both the overall and country-level prevalence estimates were calculated as weighted estimates to account for any differences between the actual and planned sampling distribution of participants by healthcare setting (primary care:specialist care).

The study sample was characterized by demographics, clinical parameters, CV medication, and GLA usage, with particular reference to GLAs with demonstrated CV benefit, presented for the overall study sample and separately for the CVD group and non-CVD group; data were not weighted.

#### Post hoc* analyses*

The number of affected vascular areas among those with CVD was calculated and stratified by gender. Three vascular areas were analyzed, defined as coronary, cerebrovascular and peripheral.

To explore whether differences in estimated CVD prevalence between countries could be partially or fully explained by differences in demographic and clinical characteristics between the country samples, logistic regression models were used to calculate prevalence odds ratios (PORs) for CVD in each country using the overall CAPTURE study sample as the reference. The models were as follows: (1) crude; (2) adjusted for age and gender; and (3) additionally adjusted for statistically significant clinical parameters identified via backwards selection (age, gender, diabetes duration, body mass index [BMI], glycated hemoglobin [HbA_1c_], low-density lipoprotein cholesterol, high-density lipoprotein cholesterol, smoking status, hypertension, nephropathy, neuropathy, and retinopathy). Parameters with a high proportion of missing data (albuminuria, estimated glomerular filtration rate [eGFR] and physical activity) were not considered (Additional file 1: Table S2). Any missing data for the included parameters were imputed for each country separately using fully conditional specification. Sensitivity analyses were conducted (1) without imputation of missing data for participants with complete covariate information and (2) including eGFR as a clinical parameter (both with and without imputation of missing data). Further details on the logistic regression analyses are available in Additional file 1: Methods S2.

Due to the large T2D population compared to the other study countries, China would have a large influence on the overall weighted CVD prevalence estimates. As such, a sensitivity analysis was conducted where the prevalence calculations were repeated for all countries excluding China. These estimates were also calculated as weighted estimates to account for the size of the diabetes population of the 12 remaining countries [26].

In order to place the CAPTURE data in the context of two recent CV outcome trials, exploratory analyses assessed the number of participants with high CVD risk in the non-CVD group who were using a GLA with demonstrated CV benefit. Participants with high CVD risk were identified in the non-CVD group as satisfying relevant criteria from the REWIND [27] or DECLARE-TIMI 58 [28] trials based on available CAPTURE data. To align as closely as possible with REWIND criteria, participants in the non-CVD group were categorized as being at high CVD risk if they were ≥ 60 years of age; were a current (any self-reported current tobacco use) or previous smoker (any self-reported historical tobacco use); and were on anti-hypertensive medication or had elevated systolic blood pressure (≥ 140 mmHg) or had elevated diastolic blood pressure (≥ 95 mmHg) [27]. To align as closely as possible with DECLARE-TIMI 58 criteria, participants in the non-CVD group were categorized as being at high CVD risk if they were ≥ 55 years of age if male or ≥ 60 years of age if female; and were either on anti-hypertensive medication or had both elevated systolic blood pressure (> 140 mmHg) and elevated diastolic blood pressure (> 90 mmHg) [28].

All statistical analyses were carried out using SAS, Version 9.4 (SAS Institute, Cary, NC, USA).

## Results

### Study sample

The overall study sample included 9823 adults with T2D (n = 4502 from primary care; n = 5321 from specialist care) (Additional file 1: Fig. S1 for study flow) who participated between December 01, 2018 and September 30, 2019. The median (interquartile range [IQR]) number of participants per site was 40 (25–57). The number and geographical distribution of study participants are presented in Additional file 1: Fig. S2. The median (IQR) age of the overall study sample was 64.0 years (56.0–71.0 years) and 45.5% of participants were female (Table 1; additional data in Additional file 1: Table S3). Most (80.4%) participants had a BMI ≥ 25 kg/m^2^, and 70.1% had diagnosed hypertension. The median (IQR) HbA_1c_ was 7.30% (6.60–8.40%) [56 mmol/mol (49–68 mmol/mol)] and diabetes duration was 10.7 years (5.6–17.9 years).

**Table 1:** Demographic and clinical characteristics of the CAPTURE study sample overall and stratified by CVD status

Characteristic	Overall N = 9823		By CVD status			
			CVD n = 3582		Non-CVD n = 6241	
	n	Data	n	Data	n	Data
Female, n (%)	9823	4465 (45.5)	3582	1388 (38.7)	6241	3077 (49.3)
Age, years, median [IQR]	9823	64.0 [56.0–71.0]	3582	68.0 [61.0–75.0]	6241	62.0 [54.0–69.0]
Race, n (%)	9822		3581		6241	
White		6487 (66.0)		2558 (71.4)		3929 (63.0)
Asian		2133 (21.7)		718 (20.1)		1415 (22.7)
Black or African American		158 (1.6)		66 (1.8)		92 (1.5)
Other		1044 (10.6)		239 (6.7)		805 (12.9)
Diabetes duration, years, median [IQR]	9811	10.7 [5.6–17.9]	3577	13.0 [7.2–20.0]	6234	9.8 [4.8–15.9]
HbA_1c_, %, median [IQR]	9104	7.30 [6.60–8.40]	3289	7.40 [6.60–8.50]	5815	7.30 [6.50–8.30]
HbA_1c_, mmol/mol, median [IQR]	9104	56 [49–68]	3289	57 [49–69]	5815	56 [48–67]
FPG, mmol/L, median [IQR]	8204	7.60 [6.30–9.38]	2924	7.60 [6.21–9.43]	5280	7.60 [6.33–9.32]
Body weight, kg, median [IQR]	9742	79.3 [68.7–92.0]	3550	79.8 [69.0–92.0]	6192	79.0 [68.3–92.0]
BMI, kg/m^2^, median [IQR]	9611	29.0 [25.8–33.1]	3514	28.9 [25.7–33.1]	6097	29.1 [25.8–33.1]
Systolic blood pressure, mmHg, median [IQR]	9618	130.0 [120.0–140.0]	3531	130.0 [120.0–142.0]	6087	130.0 [120.0–140.0]
Diastolic blood pressure, mmHg, median [IQR]	9616	78.0 [70.0–82.0]	3529	76.0 [70.0–81.0]	6087	80.0 [70.0–83.0]
Total cholesterol, mmol/L, median [IQR]	8272	4.34 [3.68–5.14]	3001	4.05 [3.39–4.82]	5271	4.51 [3.83–5.26]
LDL cholesterol, mmol/L, median [IQR]	8090	2.39 [1.81–3.08]	2924	2.12 [1.62–2.77]	5166	2.54 [1.98–3.19]
HDL cholesterol, mmol/L, median [IQR]	7965	1.15 [0.98–1.40]	2907	1.11 [0.93–1.32]	5058	1.18 [0.99–1.42]
Triglyceride, mmol/L, median [IQR]	8466	1.60 [1.13–2.27]	3082	1.61 [1.14–2.30]	5384	1.59 [1.13–2.25]
eGFR, mL/min/1.73 m^2^, n (%)	7923		2888		5035	
> 89		2746 (34.7)		707 (24.5)		2039 (40.5)
> 59–89		3512 (44.3)		1293 (44.8)		2219 (44.1)
> 29–59		1450 (18.3)		757 (26.2)		693 (13.8)
≤ 29		215 (2.7)		131 (4.5)		84 (1.7)
Albuminuria^a^, n (%)	6482		2433		4049	
Normal–mildly increased		4338 (66.9)		1396 (57.4)		2942 (72.7)
Microalbuminuria		1607 (24.8)		774 (31.8)		833 (20.6)
Macroalbuminuria		537 (8.3)		263 (10.8)		274 (6.8)
Medical history of hypertension, yes, n (%)	9643	6756 (70.1)	3522	2918 (82.9)	6121	3838 (62.7)
Familial hypercholesterolemia, yes, n (%)	6634	676 (10.2)	2342	246 (10.5)	4292	430 (10.0)
Retinopathy, n (%)	9818		3578		6240	
Yes		1455 (14.8)		725 (20.3)		730 (11.7)
Yes (referred by participant)		399 (4.1)		144 (4.0)		255 (4.1)
No		7964 (81.1)		2709 (75.7)		5255 (84.2)
Nephropathy, n (%)	9818		3579		6239	
Yes		1771 (18.0)		917 (25.6)		854 (13.7)
Yes (referred by participant)		337 (3.4)		128 (3.6)		209 (3.3)
No		7710 (78.5)		2534 (70.8)		5176 (83.0)
Neuropathy, n (%)	9817		3577		6240	
Yes		1774 (18.1)		867 (24.2)		907 (14.5)
Yes (referred by participant)		459 (4.7)		168 (4.7)		291 (4.7)
No		7584 (77.3)		2542 (71.1)		5042 (80.8)
Smoking status, n (%)	9725		3547		6178	
Current		1322 (13.6)		465 (13.1)		857 (13.9)
Previous		2613 (26.9)		1268 (35.7)		1345 (21.8)
Never		5790 (59.5)		1814 (51.1)		3976 (64.4)
Duration of smoking^b^, years, median [IQR]	3733	28.0 [15.0–39.0]	1646	30.0 [20.0–40.0]	2087	25.0 [15.0–35.0]
Physical activity^c^, days per week, n (%)	7492		2763		4729	
0–1		3599 (48.0)		1515 (54.8)		2084 (44.1)
2–3		1613 (21.5)		497 (18.0)		1116 (23.6)
4–5		883 (11.8)		264 (9.6)		619 (13.1)
6–7		1397 (18.6)		487 (17.6)		910 (19.2)

To convert the values for glucose to mg/dL, divide by 0.0555. To convert the values for cholesterol to mg/dL, divide by 0.0259. To convert the values for triglycerides to mg/dL, divide by 0.0113. Data that were missing from the medical record but verbally confirmed by a participant were coded as ‘referred by participant’. Data were not weighted

^a^Defined as: normal–mildly increased, urinary excretion < 30 mg/24 h or UACR < 30 mg/g; microalbuminuria, urinary excretion 30–299 mg/24 h or UACR 30–299 mg/g; macroalbuminuria, urinary excretion ≥ 300 mg/24 h or UACR ≥ 300 mg/g

^b^Only applies to participants categorized as current or previous smokers

^c^Days with ≥ 30 min of moderate activity

*BMI* body mass index*, CVD* cardiovascular disease, *eGFR* estimated glomerular filtration rate, *FPG* fasting plasma glucose, *HbA*_*1c*_ glycated hemoglobin, *HDL* high-density lipoprotein, *IQR* interquartile range, *LDL* low-density lipoprotein, *UACR* urinary albumin to creatinine ratio

### CVD prevalence

**Fig. 1:**
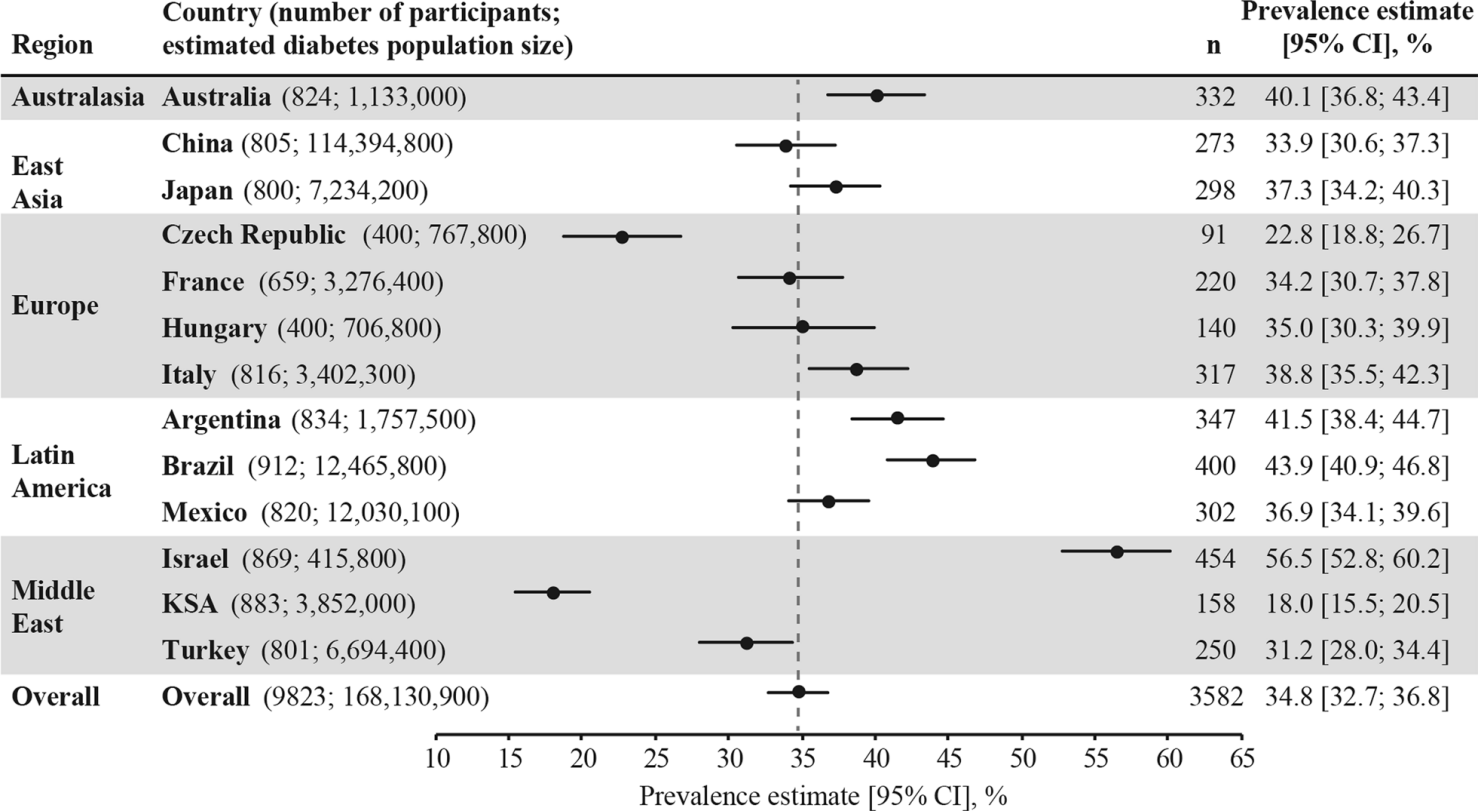
Weighted CVD prevalence in people with type 2 diabetes across the 13 countries. Data presented overall and by country. Overall prevalence estimate (across the 13 countries) calculated as a weighted estimate to account for the size of the diabetes population of each country [26] and represented by the grey dotted line. Both the overall and country-level prevalence estimates were weighted by the sampling of participants by healthcare setting, if it was different from as planned. n numbers are the crude number of participants with CVD (i.e. they were not weighted). *CI* confidence interval, *CVD* cardiovascular disease, *KSA* Kingdom of Saudi Arabia, *n* number of participants with CVD

**Fig. 2:**
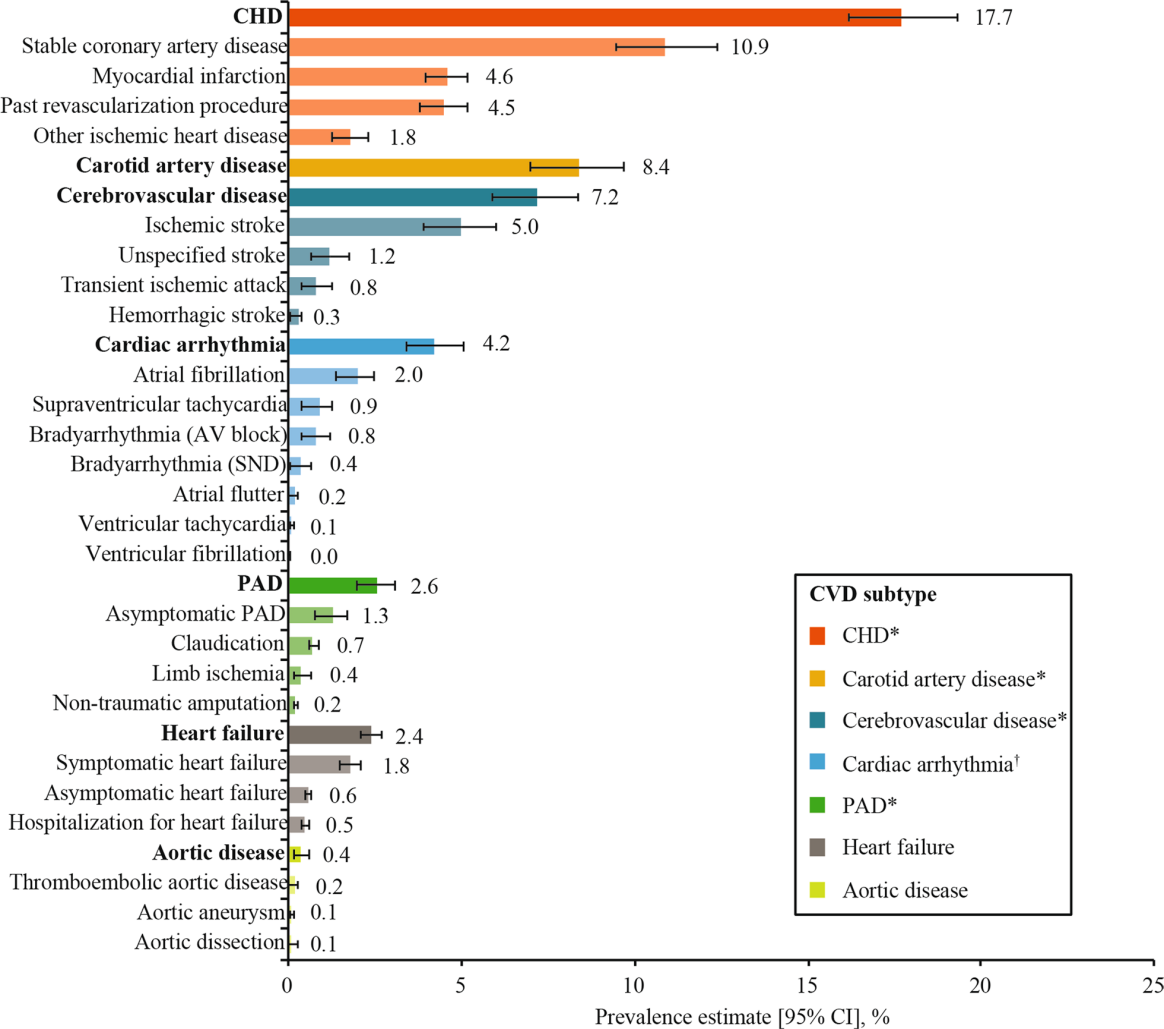
Overall weighted CVD prevalence in people with type 2 diabetes by CVD subtype and diagnosis. Data are overall prevalence estimates (95% CI), which were calculated as weighted estimates to account for the size of the diabetes population of each country [26] and the sampling of participants by healthcare setting, if it was different from as planned. Along the y-axis, CVD subtypes are in bold font, while the diagnoses contributing to each subtype are in plain font. Diagnoses are not mutually exclusive; one participant may have multiple diagnoses. ^*^Categorized as ASCVD. ^†^Included conduction abnormalities. *ASCVD* atherosclerotic CVD, *AV* atrioventricular, *CHD* coronary heart disease, *CI* confidence interval, *CVD* cardiovascular disease, *PAD* peripheral artery disease, *SND* sinus node dysfunction

Among the 9823 study participants, over one third (n = 3582; 36.5%) had established CVD, with a weighted CVD prevalence estimated at 34.8% (95% CI 32.7–36.8%) across the 13 countries. CVD prevalence was lowest in the Kingdom of Saudi Arabia (18.0%) and highest in Israel (56.5%) (Fig. 1). Most (85.8%; n = 3074) cases of CVD were atherosclerotic, with the weighted ASCVD prevalence estimated at 31.8% (95% CI 29.7–33.8%) across the 13 countries. The weighted prevalence of CVD subtypes and diagnoses are presented in Fig. 2 and Additional file 1: Table S4. The most prevalent weighted CVD subtypes were CHD (17.7%), carotid artery disease (8.4%), and cerebrovascular disease (7.2%). The overall weighted prevalence of heart failure was 2.4% (95% CI 2.1–2.7%), with a wide range from 0.2% in China to 14.5% in Israel (Additional file 1: Fig. S3). Overall, the weighted prevalence of CVD was similar in participants from primary versus specialist care (35.6% vs 34.6%) (not statistically analyzed).

In post hoc analyses that excluded China from the overall prevalence estimates, the weighted CVD prevalence was 36.6% (95% CI 35.4–37.7%) and heart failure prevalence was 6.9% (95% CI 6.3–7.6%) across the 12 remaining countries.

### Characteristics of the study sample by CVD status

In comparison with the non-CVD group, the CVD group was older (median age 68.0 years vs 62.0 years), had a higher proportion of males (61.3% vs 50.7%), and a longer duration of diabetes (median duration 13.0 years vs 9.8 years) (Table 1; not statistically analyzed). In comparison with the non-CVD group, a higher proportion of participants in the CVD group had diagnosed hypertension (82.9% vs 62.7%), renal dysfunction (microalbuminuria, 31.8% vs 20.6%; macroalbuminuria, 10.8% vs 6.8%; eGFR ≤ 59 mL/min/1.73 m^2^, 30.7% vs 15.4%), were current or previous smokers (48.9% vs 35.6%), and reported low physical activity (0–1 day of physical activity per week, 54.8% vs 44.1%) (Table 1; additional data in Additional file 1: Table S3). In contrast, median serum low-density lipoprotein cholesterol concentrations were lower in the CVD group versus the non-CVD group (2.12 mmol/L vs 2.54 mmol/L). The proportion of participants with microvascular complications was higher in the CVD group compared with the non-CVD group (retinopathy: 24.3% vs 15.8%; nephropathy: 29.2% vs 17.0%; neuropathy: 29.0% vs 19.2%).

### GLAs by CVD status

**Fig. 3:**
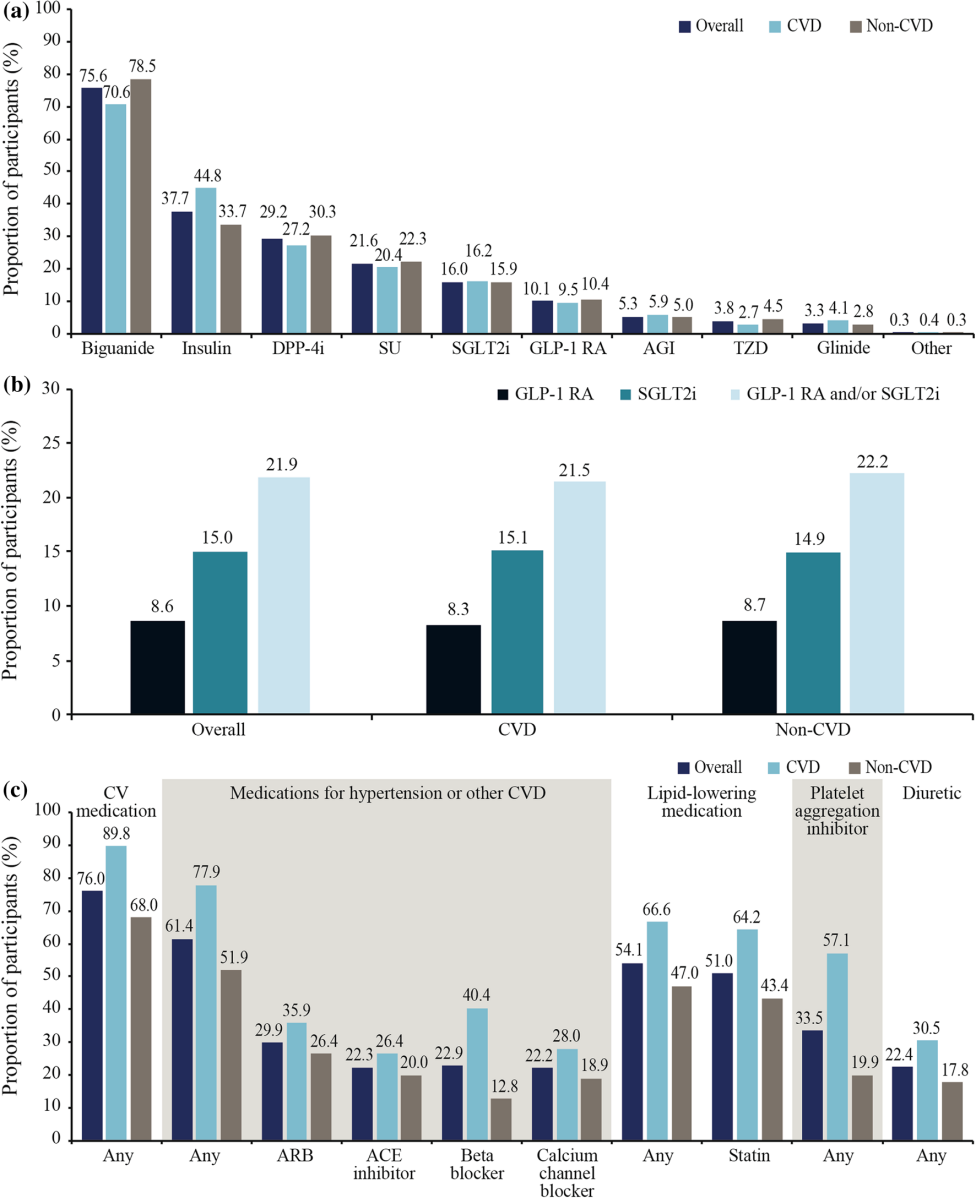
Use of **a** GLAs, **b** GLAs with demonstrated CV benefit and **c** selected CV medications. Data are % and were not weighted. Data are presented for the CAPTURE study sample overall and stratified by CVD status. In **b**, GLAs with demonstrated CV benefit were defined *per* 2020 American Diabetes Association guidelines [19] as GLP-1 RAs: dulaglutide, liraglutide, and semaglutide; and SGLT2is: canagliflozin, dapagliflozin, and empagliflozin. *ACE* angiotensin-converting enzyme, *AGI* alpha glucosidase inhibitor, *ARB* angiotensin II receptor blocker, *CV* cardiovascular, *CVD* cardiovascular disease, *DPP-4i* dipeptidyl peptidase-4 inhibitor, *GLA* blood glucose-lowering agent, *GLP-1 RA* glucagon-like peptide-1 receptor agonist, *SGLT2i* sodium-glucose co-transporter-2 inhibitor, *SU* sulfonylurea, *TZD* thiazolidinedione

In total, 96.6% of the overall study sample were receiving at least one GLA from any class. Biguanides were less frequently prescribed in the CVD group than the non-CVD group (70.6% vs 78.5%), whereas insulin use was more common in the CVD group than in the non-CVD group (44.8% vs 33.7%) (Fig. 3a). In total, 21.9% of participants were prescribed a GLA with demonstrated CV benefit, and this was similar in the CVD and non-CVD groups (21.5% vs 22.2%) (Fig. 3b). SGLT2is were more frequently used than GLP-1 RAs (15.0% vs 8.6%) in the overall study sample, with a similar use of both therapeutic classes across the CVD and non-CVD groups.

In exploratory analyses, 2051 and 2498 participants from the non-CVD group were classified as having high CVD risk using some of the REWIND and DECLARE-TIMI 58 criteria, respectively. In total, 22.2% (n = 456) of participants with high CVD risk according to REWIND criteria and 21.6% (n = 540) of participants with high CVD risk according to DECLARE-TIMI 58 criteria were using a GLA with demonstrated CV benefit.

### CV medications by CVD status

Overall, 7461 participants (76.0%) were receiving any CV medication and it was more common in the CVD group than in the non-CVD group (89.8% vs 68.0%), with the same pattern across CV medication classes (Fig. 3c; not statistically analyzed). In the CVD group, statins were the most frequently utilized CV medications (64.2%), followed by beta-blockers (40.4%), acetylsalicylic acid (39.0%), angiotensin II receptor blockers (ARBs; 35.9%), calcium channel blockers (28.0%), and angiotensin-converting enzyme (ACE) inhibitors (26.4%). In the non-CVD group, statin use also predominated (43.4%), followed by ARBs (26.4%), ACE inhibitors (20.0%), acetylsalicylic acid (19.1%), calcium channel blockers (18.9%), and beta-blockers (12.8%). Among participants at high CVD risk in the non-CVD group, statins were most frequently used (43.4%), followed by ARBs (30.3%), calcium channel blockers (24.3%), ACE inhibitors (23.6%), acetylsalicylic acid (23.3%), and beta-blockers (14.0%).

### Vascular areas affected according to gender

When analyzing the number of affected vascular areas in participants with CVD, data were available for 1134 women and 1940 men. The proportion of participants with one vascular area affected was similar among men and women, although men were more likely to have more vascular areas involved than women (Additional file 1: Fig. S4).

### PORs for CVD adjusted for age, gender, and clinical parameters

Differences in age and gender explained some of the variation in CVD prevalence between countries, as PORs in model 1 (age- and gender-adjusted) were closer to 1.00 for some countries (Argentina, Australia, China, Israel, Kingdom of Saudi Arabia, and Turkey) than in the unadjusted model (Additional file 1: Fig. S5). Adjustment for additional clinical parameters (model 2) appeared to explain further variation in CVD prevalence between countries observed in the unadjusted model, with PORs closer to 1.00 for all countries except China, France, and Mexico. However, there were still significant differences in CVD prevalence for some countries in comparison with the overall study sample after further adjustment, specifically lower odds of CVD for Kingdom of Saudi Arabia, Czech Republic, and France, in addition to higher odds of CVD for Israel, Brazil, and China (p < 0.05 for all comparisons; Additional file 1: Fig. S5). Findings from these adjusted analyses were consistent with sensitivity analyses without imputation of missing data (Additional file 1: Fig. S6) and including eGFR (both with and without imputation of missing data) (Additional file 1: Fig. S7).

## Discussion

The overall weighted prevalence of CVD ascertained using standardized methodology in the present multinational study of adults with T2D from primary or specialist care settings was estimated at 34.8%. ASCVD accounted for most (85.8%) of this disease burden, with stable coronary artery disease, carotid artery disease, and stroke the major components.

The 2019 CAPTURE findings are in accordance with those reported by Einarson and colleagues [16] in their systematic literature review of 57 studies from 25 countries involving over 4.5 million adults with T2D conducted between 1987 and 2015, which estimated a CVD prevalence of 32.2% [16]. Our weighted prevalence estimate for ASCVD was also similar to that in the systematic review (31.8% vs 29.1%, respectively) [16]. Other available comparative data are from individual countries not included in CAPTURE. Nevertheless, our CVD prevalence estimate aligns with CVD data linked to hospital admissions for nearly 250,000 Scottish patients with T2D (32.5%) [14]. However, it is higher than that reported in a primary care survey of over 17,000 Danish patients with T2D (21.4%) [11], and our weighted ASCVD prevalence estimate is lower than the 45.2% reported in a cross-sectional study of over 1.2 million adults with T2D from a US claims database in 2015 [10]. In addition to potential geographical differences, direct comparisons between studies are complicated by heterogeneity in factors such as participant selection, study design, timing, and methods of CVD ascertainment. Nevertheless, the CAPTURE data, collected using standardized methodology across 13 countries, make progress in addressing the need for more uniform epidemiological data relating to the global CVD burden [29].

In the present study, post hoc analyses were carried out to identify explanations for the between-country variation in CVD estimates. Differences in age and gender accounted for some of the observed variation between countries, while adjustment for additional clinical parameters further attenuated individual country differences from the pooled estimate. However, even after additional adjustment for potentially confounding parameters, there were significantly lower odds of CVD in the Kingdom of Saudi Arabia, Czech Republic, and France, contrasting with significantly higher odds in Israel, Brazil, and China, as compared with the overall CAPTURE study sample. These between-country differences might reflect variation in the sites selected, genetic and/or lifestyle factors, healthcare accessibility and delivery, CV medication use, medical record characteristics, CVD screening practices, and even competing risk of death from non-diabetes-related causes [30, 31], factors that were not directly addressed in CAPTURE. Our country-level CVD prevalence estimates are similar to those reported by Einarson and colleagues [16] in their systematic literature review, wherein the prevalence in China was 33.9% in CAPTURE vs 28.4% in the systematic literature review. However, there was variation among the CVD estimates for the other countries in CAPTURE with outlying odds when compared with available data (Saudi Arabia: 18.0% vs 30.0%; France: 34.2% vs 53.9%; Brazil: 43.9% vs 27.5%, all for CAPTURE vs the systematic literature review [16], respectively). A detailed exploration of outliers was beyond the scope of the present study. With a large diabetes population, China accounted for most of the weighting and may have substantially influenced the results. However, post hoc analyses excluding China found that the overall weighted CVD prevalence was only marginally higher (1.8 percentage points) for the remaining 12 countries, despite a more pronounced influence of China on the weighted heart failure prevalence, which was 4.2 percentage points higher for the remaining 12 countries. This large variation in heart failure prevalence may be attributed to underreporting and missed diagnoses, which may vary considerably between countries, depending on their screening and diagnostic capabilities [32].

Motivated by evidence that ASCVD is largely preventable, many countries are starting to implement policies and practices that aim to decrease this burden in people at high risk, including those with T2D [29, 33]. Effective action to reduce the global burden of CVD in people with T2D requires reliable data on prevalence, risk factors, medication use, and the barriers to prevention and treatment [34]. Our findings indicate that fewer than one in four adults with T2D and established CVD use a GLA with demonstrated CV benefit. In 2015, when relatively few relevant CV outcome trial data were available, an understandably lower use of GLP-1 RAs (7.9%) and SGLT2is (8.8%) was reported in over 500,000 US adults with T2D and established ASCVD [10].

Additionally, our findings indicate that a relatively low proportion of adults with T2D without established CVD but at high CVD risk in CAPTURE – approximately 20% – used a GLA with demonstrated CV benefit. This has potential clinical implications as REWIND found a non-significant trend to fewer major adverse CV events with dulaglutide versus placebo in patients with CV risk factors but without CVD (hazard ratio [HR] 0.87, 95% CI 0.74–1.02) [27], and DECLARE-TIMI 58 reported a non-significant trend towards a lower rate of a composite of CV death or hospitalization for heart failure with dapagliflozin versus placebo in the same subgroup (HR 0.84, 95% CI 0.67–1.04) [28].

The proportions of patients using GLAs with demonstrated CV benefit may change with the implementation and influence of recent updates to diabetes/cardiology guidelines that now recommend a GLP-1 RA or SGLT2i with demonstrated CV benefit as first- or second-line GLA in people with T2D and established CVD or at high/very high CVD risk [5–7]. The future impact of these updates on real-world clinical practice will be of interest, and our contemporary data provide a benchmark against which relevant trends can be monitored. Indeed, regional differences in diabetes and cardiology treatment guidelines, as well as approvals and reimbursement of individual medications including GLAs, are also likely to influence the use of GLAs with CV benefit at a country level. For example, in Brazil, physicians have the ability to prescribe any class of GLA and are limited only by financial considerations. In France, SGLT2is where not approved for use at the time of the CAPTURE study, and even today, GPs have the ability to prescribe GLP-1 RAs but are only permitted to renew prescriptions of SGLT2is after they have been initiated by a specialist. In the Kingdom of Saudi Arabia, the availability of GLP-1 RAs and SGLT2i GLAs is dependent on the purchasing decisions, policies and prescribing privileges in individual governmental hospitals.

A treatment gap exists for other evidence-based therapies. For example, a smaller than recommended proportion of participants with established CVD in CAPTURE were using statins (64.2%) or acetylsalicylic acid (39.0%) [6, 35]. In patients with known CVD, acetylsalicylic acid reduces CV events, with the benefits outweighing the risk of major bleeding [36]. Statins have also been found to be effective in reducing CV events in patients with or without established CVD [37]. The CAPTURE data highlight the potential for improved use of non-glycemic CV pharmacotherapy for people with T2D and CV risk factors. Notably, glycemia, as assessed by HbA_1c_, was relatively well controlled in the overall CAPTURE study sample (median 7.3% [56 mmol/mol]). This is in alignment with the HbA_1c_ levels reported by similar studies that estimated CVD prevalence in patients with T2D in primary care (mean 6.9% [52 mmol/mol]) [11] and admitted to hospital (median 7.2% [55 mmol/mol]) [14], albeit in other European settings.

The CAPTURE study has several strengths. It was cross-sectional and multinational in design, with consistent methodology for data collection across different healthcare systems through use of a standardized electronic case report form. Participants were recruited from both primary and specialist care. Broad inclusion criteria were used to ensure that the study sample was as representative as possible of the general adult T2D population, with implications for the generalizability of the findings. As evidenced by key demographic and clinical characteristics, the participants spanned a wide spectrum of disease. Our findings provide contemporary prevalence estimates for several non-US countries where up-to-date data were limited or absent [16]. Furthermore, our findings may provide valuable background data for local healthcare payers and policy makers to assist with evaluating strategies to reduce CVD risk in adults with T2D. The application of our findings to the design of CV outcome trials in the study countries could enhance the generalizability of their results by informing trial entry criteria and aiming to increase the representative nature of the trial sample compared with the general T2D population.

The present study has limitations. It is possible that our prevalence data are overestimates, as there may be a tendency for people with complications to consult their healthcare provider more frequently than the general T2D population. This form of ascertainment bias could explain why the CVD prevalence was similar in primary and specialist care. Additionally, very ill people with relatively high rates of complications including CVD may have been unable to attend routine healthcare visits. We cannot exclude the possibility of consent bias, a potential limitation of any study requiring participants’ consent for inclusion, in that individuals willing to participate in the study may not have been fully representative of the general population of patients with T2D. Medication use may have been influenced by country-specific guidelines for management of patients with T2D and between-country differences in approvals and reimbursement of individual medications and GLA classes. Furthermore, our study was non-interventional and did not mandate screening for, or adjudication of, the presence of complications. Our findings relied on the clinical capabilities and documentation specific to each participant’s healthcare setting. The non-interventional nature of CAPTURE meant that some participants may have had undiagnosed or misdiagnosed CVD, particularly in clinics or countries where relevant investigations were not recommended, there was a lack of diagnostic capability, or where medical records were fragmented or incomplete. As it was important to identify a study population representative of patients with T2D regularly attending primary and/or secondary diabetes centers across the different countries and guarantee accuracy in data collection, the choice of participating sites in primary and specialist care was based on recommendations from personnel with knowledge of the local health system who were employed by the sponsor rather than real-world country-specific data, with the possibility of selection bias. However, final participating sites were selected by the contract research organization (and approved by personnel employed by the sponsor) to optimize the accuracy of CVD data collection.

## Conclusions

CAPTURE found that approximately one in three adults with T2D had established CVD. ASCVD accounted for most of this burden, with stable coronary artery disease, carotid artery disease, and stroke being the major contributors. Most participants with CVD were not managed according to the most recent diabetes and cardiology guidelines, implying potential scope for reducing the excess risk through evidence-based interventions. Our data provide a benchmark against which trends can be monitored in order to evaluate the implementation of recent international guidelines.

## Supplementary Information

**Additional file 1. **Additional methods, data and analyses and full list of CAPTURE study investigators/collaborators.

## Data Availability

The datasets used and/or analyzed during the current study are available from the corresponding author on reasonable request.

## Abbreviations

ACE: Angiotensin-converting enzyme
ARB: Angiotensin II receptor blocker
ASCVD: Atherosclerotic cardiovascular disease
BMI: Body mass index
CHD: Coronary heart disease
CI: Confidence interval
CV: Cardiovascular
CVD: Cardiovascular disease
eGFR: Estimated glomerular filtration rate
GLA: Blood glucose-lowering agent
GLP-1 RA: Glucagon-like peptide-1 receptor agonist
HbA_1c_: Glycated hemoglobin
HR: Hazard ratio
IQR: Interquartile range
PAD: Peripheral artery disease
POR: Prevalence odds ratio
SGLT2i: Sodium-glucose co-transporter-2 inhibitor
