# Supplementary material for: CAPTURE: a multinational, cross-sectional study of cardiovascular disease prevalence in adults with type 2 diabetes across 13 countries
Source: Cardiovasc Diabetol. 2021 Jul 27;20:154. doi: 10.1186/s12933-021-01344-0 (PMC8317423; doi:10.1186/s12933-021-01344-0)
Supplement: Supplementary file 1 — Additional file 1. Additional methods, data and analyses and full list of CAPTURE study investigators/collaborators. [file 12933_2021_1344_MOESM1_ESM.pdf]

CAPTURE: a multinational, cross-sectional study of cardiovascular disease prevalence in adults with type 2 diabetes across 13 countries

Ofri Mosenzon, Abdullah Alguwaihes, Jose Luis Arenas Leon, Fahri Bayram, Patrice Darmon, Timothy M.E. Davis, Guillermo Dieuzeide, Kirsten T. Eriksen, Tianpei Hong, Margit S. Kaltoft, Csaba Lengyel, Nicolai A. Rhee, Giuseppina T. Russo, Shinichiro Shirabe, Katerina Urbancova, Sergio Vencio, on behalf of the CAPTURE study investigators

ADDITIONAL MATERIAL

Methods S1. **List of cardiovascular (CV) medications in the CAPTURE study**

Alirocumab, amlodipine, atenolol, atorvastatin, bisoprolol, candesartan, captopril, carvedilol, clopidogrel, digoxin, dronedarone, edoxaban, enalapril, evolocumab, ezetimibe, fenofibrate, furosemide, glyceryl trinitrate, hydrochlorothiazide, indapamide, irbesartan, isosorbide mononitrate, ivabradine, lisinopril, losartan, metoprolol, nebivolol, nicotinic acid, nifedipine, perindopril, prasugrel, pravastatin, propranolol, ramipril, ranolazine, rosuvastatin, sacubitril, simvastatin, sotalol, spironolactone, telmisartan, ticagrelor, torasemide, trandolapril, valsartan and warfarin.

Methods S2. Additional methodological details for the multivariable logistic regression analyses

Candidate covariates

The candidate covariates were country, gender, age, diabetes duration, body mass index (BMI), glycated hemoglobin (HbA_{1c}), low-density lipoprotein (LDL) cholesterol, high-density lipoprotein (HDL) cholesterol, smoking status, hypertension, nephropathy, neuropathy, and retinopathy. Some parameters with a high proportion of missing data were not considered as candidate covariates (albuminuria, estimated glomerular filtration rate [eGFR], and physical activity; refer to Table S2). Additional sensitivity analyses were performed including eGFR as a candidate covariate.

Main analysis

Cardiovascular disease (CVD) prevalence odds ratios (PORs) were estimated in SAS PROC LOGISTIC using multivariable logistic regression models with CVD (yes/no) as dependent variable and the following covariates: model 0: country (crude bivariable model); model 1: country, gender, and age; model 2: country, gender, age, diabetes duration, BMI, HbA_{1c}, LDL cholesterol, HDL cholesterol, smoking status, hypertension, nephropathy, neuropathy, and retinopathy. The overall CAPTURE study sample was included as the reference group, with data for participants with

available covariate information merged with the dataset (i.e. doubling the number of observations). Missing data for LDL cholesterol were imputed using the following formulae (when data for non-HDL cholesterol were available):

LDL cholesterol (mg/dL) = non-HDL cholesterol (mg/dL) minus 30

LDL cholesterol (mmol/L) = LDL cholesterol (mg/dL) divided by 38.61

Other missing data were imputed by fully conditional specification. Missing data for LDL and HDL cholesterol in 110 participants from France could not be imputed. The final (reduced) model 2 (described above) was chosen via backwards selection method starting with a full (saturated) model including all clinically important demographic and clinical characteristics, which are those moderately associated with CVD prevalence in bivariable logistic regression (significance level of $p < 0.25$) and without multicollinearity (variance inflation factor ≤ 5) from the list of candidate covariates. Backwards selection was performed with a significance threshold of $p < 0.05$.

Sensitivity analyses

Sensitivity analyses were performed where the analysis (described above) was repeated (1) without imputation of missing demographic and clinical data (analyses performed for participants with complete covariate information); and (2) including eGFR as a candidate covariate with and without imputation of missing demographic and clinical data.

Table S1. Definition of CVD diagnoses in the CAPTURE study

CVD subtype	Accepted diagnoses	Further optional details
Cerebrovascular disease	Ischemic stroke	–
	Hemorrhagic stroke	–
	Unspecified stroke	–
	Transient ischemic attack	–
CHD	Myocardial infarction	–
	Stable coronary artery disease	Also referred to as angina pectoris
	Other ischemic heart disease	–
	Past revascularization procedure	–
Heart failure	Symptomatic heart failure	NYHA group II–IV or unknown; LVEF (%) < 40, 40–< 50, ≥ 50%, or unknown
	Asymptomatic heart failure	NYHA group I with LVEF (%) < 40, 40–< 50, ≥ 50%, or unknown
	Hospitalization for heart failure	–
Cardiac arrhythmia and conduction abnormalities	Atrial fibrillation	–
	Atrial flutter	–
	Ventricular tachycardia	–
	Supraventricular tachycardia	–
	Ventricular fibrillation	–
	Bradyarrhythmia	Sinus node dysfunction or atrioventricular block
Aortic disease	Aortic dissection	–
	Aortic aneurysm	–
	Thromboembolic aortic disease	–
PAD	Asymptomatic PAD	Low ankle-branchial index (< 0.90) or pulse abolition
	Claudication	–
	Limb ischemia	–
	Non-traumatic amputation	–
Carotid artery disease	Carotid artery disease	–

CHD coronary heart disease, *CVD* cardiovascular disease, *LVEF* left ventricular ejection fraction, *NYHA* New York Heart Association functional classification, *PAD* peripheral artery disease

Table S2. Distribution of missing data for demographic and clinical parameters considered as potential variables in the multivariable logistic regression analyses

Country n	Age	Sex	BMI	Hypertension	eGFR	Albuminuria	Physical activity	Smoking status	Diabetes duration	HbA_{1c}	LDL cholesterol	HDL cholesterol
Argentina (n = 834)	0	0	0	5	248	268	118	8	0	71	138	143
Australia (n = 824)	0	0	74	21	19	136	136	12	6	17	75	75
Brazil (n = 912)	0	0	5	3	238	500	73	5	0	136	202	181
China (n = 805)	0	0	17	36	512	422	282	35	0	181	275	286
Czech Republic (n = 400)	0	0	0	1	21	123	45	0	0	2	27	26
France (n = 659)	0	0	0	6	53	170	116	0	0	5	91	96
Hungary (n = 400)	0	0	0	6	17	186	114	0	0	2	148	138
Israel (n = 869)	0	0	26	15	56	97	169	27	0	23	34	36
Italy (n = 816)	0	0	0	12	129	164	365	0	0	17	123	95
Japan (n = 800)	0	0	81	16	172	302	236	11	1	6	108	124
Mexico (n = 820)	0	0	0	1	295	520	126	0	0	234	307	317
KSA (n = 883)	0	0	2	7	69	215	397	0	3	7	62	270
Turkey (n = 801)	0	0	7	51	71	238	154	0	2	18	42	71
Overall (N = 9,823)	0	0	212	180	1,900	3,341	2,331	98	12	719	1,632	1,858

Data are number of participants with missing information

BMI body mass index, *eGFR* estimated glomerular filtration rate, *HbA_{1c}* glycated hemoglobin, *HDL* high-density lipoprotein, *KSA* Kingdom of Saudi Arabia, *LDL* low-density lipoprotein

Table S3. Additional descriptive statistics summarizing the demographic and clinical characteristics of the CAPTURE study sample overall and stratified by CVD status

**Characteristic**<br>minimum; maximum<br>mean ± standard deviation	**Overall N = 9,823**		**By CVD status**			
			**CVD n = 3,582**		**Non-CVD n = 6,241**	
	**n**	**Data**	**n**	**Data**	**n**	**Data**
Age, years	9,823	18.0; 97.0<br>63.4 ± 11.4	3,582	29.0; 97.0<br>67.4 ± 10.0	6,241	18.0; 96.0<br>61.2 ± 11.5
Diabetes duration, years	9,811	0.5; 60.0<br>12.4 ± 8.6	3,577	0.5; 60.0<br>14.5 ± 9.2	6,234	0.5; 52.6<br>11.2 ± 8.1
HbA_{1c}, %	9,104	3.10; 29.00<br>7.68 ± 1.65	3,289	4.00; 18.90<br>7.73 ± 1.61	5,815	3.10; 29.00<br>7.65 ± 1.67
HbA_{1c}, mmol/mol	9,104	10; 293<br>60 ± 18	3,289	20; 183<br>61 ± 18	5,815	10; 293<br>60 ± 18
FPG, mmol/L	8,204	1.80; 33.80<br>8.32 ± 3.14	2,924	2.28; 33.57<br>8.31 ± 3.15	5,280	1.80; 33.80<br>8.33 ± 3.13
Body weight, kg	9,742	33.0; 190.0<br>81.3 ± 18.3	3,550	33.0; 178.0<br>81.5 ± 18.2	6,192	36.0; 190.0<br>81.2 ± 18.5
BMI, kg/m^2	9,611	14.5; 66.1<br>29.9 ± 5.9	3,514	14.5; 64.2<br>29.8 ± 5.8	6,097	16.2; 66.1<br>29.9 ± 6.0
Systolic blood pressure, mmHg	9,618	76.0; 251.0<br>132.2 ± 16.3	3,531	76.0; 251.0<br>132.5 ± 17.2	6,087	80.0; 226.0<br>132.1 ± 15.8
Diastolic blood pressure, mmHg	9,616	10.0; 133.0<br>76.9 ± 10.2	3,529	39.0; 129.0<br>75.5 ± 10.6	6,087	10.0; 133.0<br>77.7 ± 9.9
Total cholesterol, mmol/L	8,272	1.03; 14.80<br>4.48 ± 1.18	3,001	1.03; 12.76<br>4.21 ± 1.19	5,271	1.20; 14.80<br>4.63 ± 1.14
LDL cholesterol, mmol/L	8,090	0.20; 10.39<br>2.51 ± 0.95	2,924	0.20; 10.39<br>2.27 ± 0.94	5,166	0.39; 7.67<br>2.64 ± 0.94
HDL cholesterol, mmol/L	7,965	0.24; 5.54<br>1.21 ± 0.35	2,907	0.36; 5.54<br>1.16 ± 0.34	5,058	0.24; 3.65<br>1.24 ± 0.35
Triglyceride, mmol/L	8,466	0.25; 36.90<br>1.89 ± 1.36	3,082	0.25; 36.90<br>1.91 ± 1.42	5,384	0.30; 24.45<br>1.88 ± 1.33
Duration of smoking[a], years	3,733	0.0; 70.0<br>26.9 ± 14.4	1,646	0.0; 70.0<br>29.0 ± 14.9	2,087	0.0; 62.0<br>25.3 ± 13.8

To convert the values for glucose to mg/dL, divide by 0.0555. To convert the values for cholesterol to mg/dL, divide by 0.0259. To convert the values for triglycerides to mg/dL, divide by 0.0113. Data were not weighted

[a]Only applies to participants categorized as current or previous smokers

BMI body mass index, *CVD* cardiovascular disease, *FPG* fasting plasma glucose, *HbA_{1c}* glycated hemoglobin, *HDL* high-density lipoprotein, *LDL* low-density lipoprotein

Table S4. Overall weighted prevalence of CVD in people with type 2 diabetes by CVD subtype and diagnosis

CVD diagnosis	n	Prevalence estimate [95% CI]
Cerebrovascular disease	672	7.2 [5.9; 8.4]
Ischemic stroke	403	5.0 [3.9; 6.0]
Hemorrhagic stroke	45	0.3 [0.1; 0.4]
Stroke, unspecified	117	1.2 [0.7; 1.8]
Transient ischemic attack	141	0.8 [0.4; 1.3]
Carotid artery disease	627	8.4 [7.0; 9.7]
Coronary heart disease	2,078	17.7 [16.2; 19.3]
Myocardial infarction	930	4.6 [4.0; 5.2]
Stable coronary artery disease	639	10.9 [9.5; 12.4]
Other ischemic heart disease	524	1.8 [1.3; 2.3]
Past revascularization procedures	972	4.5 [3.8; 5.2]
Heart failure	579	2.4 [2.1; 2.7]
Symptomatic heart failure	397	1.8 [1.5; 2.1]
NYHA group II	176	1.0 [0.8; 1.2]
NYHA group III	91	0.3 [0.2; 0.4]
NYHA group IV	23	0.2 [0.0; 0.3]
NYHA unknown	104	0.3 [0.2; 0.3]
LVEF ≥ 50%	105	0.5 [0.4; 0.6]
LVEF 40–< 50%	67	0.3 [0.2; 0.4]
LVEF < 40%	106	0.4 [0.3; 0.5]
LVEF unknown	116	0.6 [0.3; 0.8]
Asymptomatic heart failure (NYHA group I)	195	0.6 [0.5; 0.7]
LVEF ≥ 50%	81	0.2 [0.1; 0.3]
LVEF 40–< 50%	36	0.1 [0.1; 0.2]

LVEF < 40%	25	0.1 [0.0; 0.1]
LVEF unknown	52	0.2 [0.1; 0.3]
Hospitalization for heart failure	170	0.5 [0.4; 0.6]
Cardiac arrhythmia and conduction abnormalities	685	4.2 [3.4; 5.1]
Atrial fibrillation	417	2.0 [1.4; 2.5]
Atrial flutter	27	0.2 [0.0; 0.3]
Supraventricular tachycardia	102	0.9 [0.4; 1.3]
Ventricular tachycardia	47	0.1 [0.1; 0.2]
Ventricular fibrillation	16	0.0 [0.0; 0.1]
Bradyarrhythmia sinus node dysfunction	34	0.4 [0.1; 0.7]
Bradyarrhythmia AV block	93	0.8 [0.4; 1.2]
Aortic disease	96	0.4 [0.2; 0.6]
Aortic dissection	14	0.1 [0.0; 0.3]
Aortic aneurysms	45	0.1 [0.1; 0.2]
Thromboembolic aortic disease	38	0.2 [0.0; 0.3]
Peripheral artery disease	489	2.6 [2.0; 3.1]
Asymptomatic peripheral artery disease	169	1.3 [0.8; 1.7]
Claudication	225	0.7 [0.6; 0.9]
Limb ischemia	84	0.4 [0.2; 0.7]
Non-traumatic amputation	75	0.2 [0.2; 0.3]

Data are overall prevalence estimates (95% CI), which were calculated as weighted estimates to account for the size of the diabetes population of each country [ref 1] and the sampling of participants by healthcare setting, if it was different from as planned. n numbers are the crude data (i.e. they were not weighted). Diagnoses are not mutually exclusive; one participant may have multiple diagnoses

AV atrioventricular, *CI* confidence interval, *CVD* cardiovascular disease, *LVEF* left ventricular ejection fraction, *n* number of participants with diagnosis, *NYHA* New York Heart Association Functional Classification

Figure S1. Participant flow through the CAPTURE study

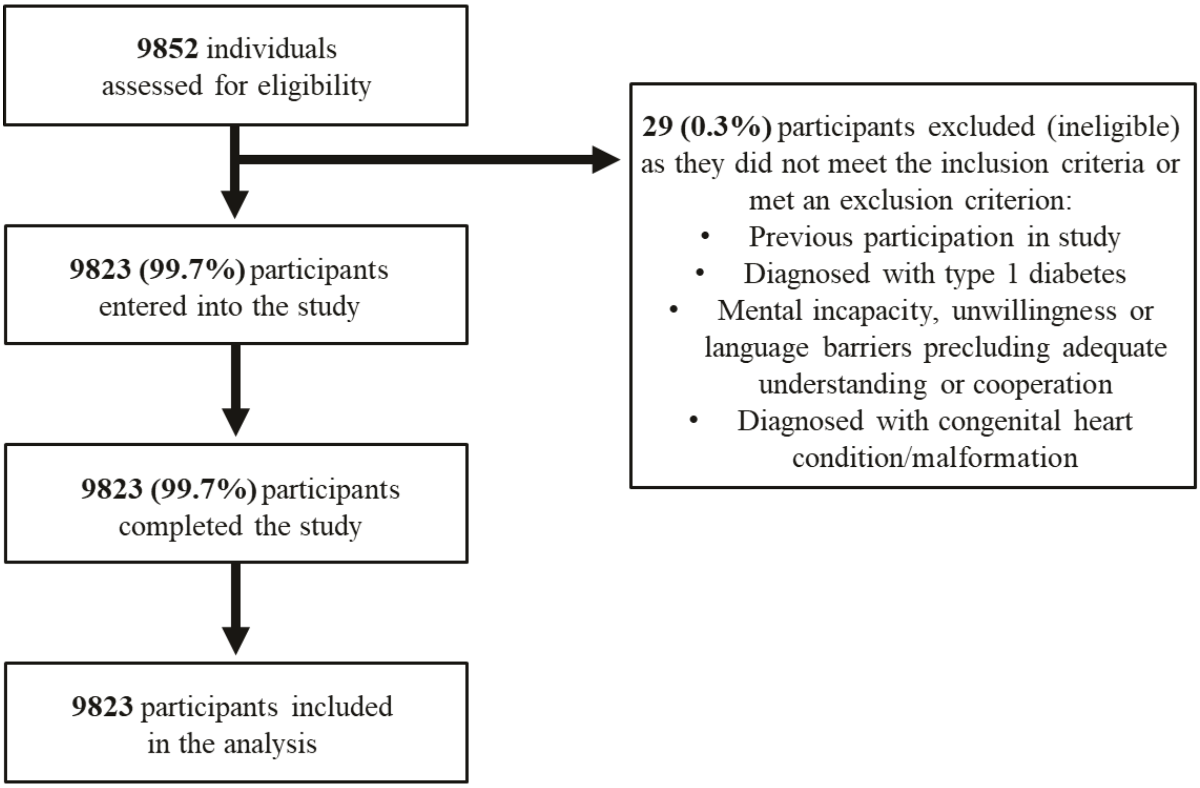

Figure S2. Global distribution of the CAPTURE study participants

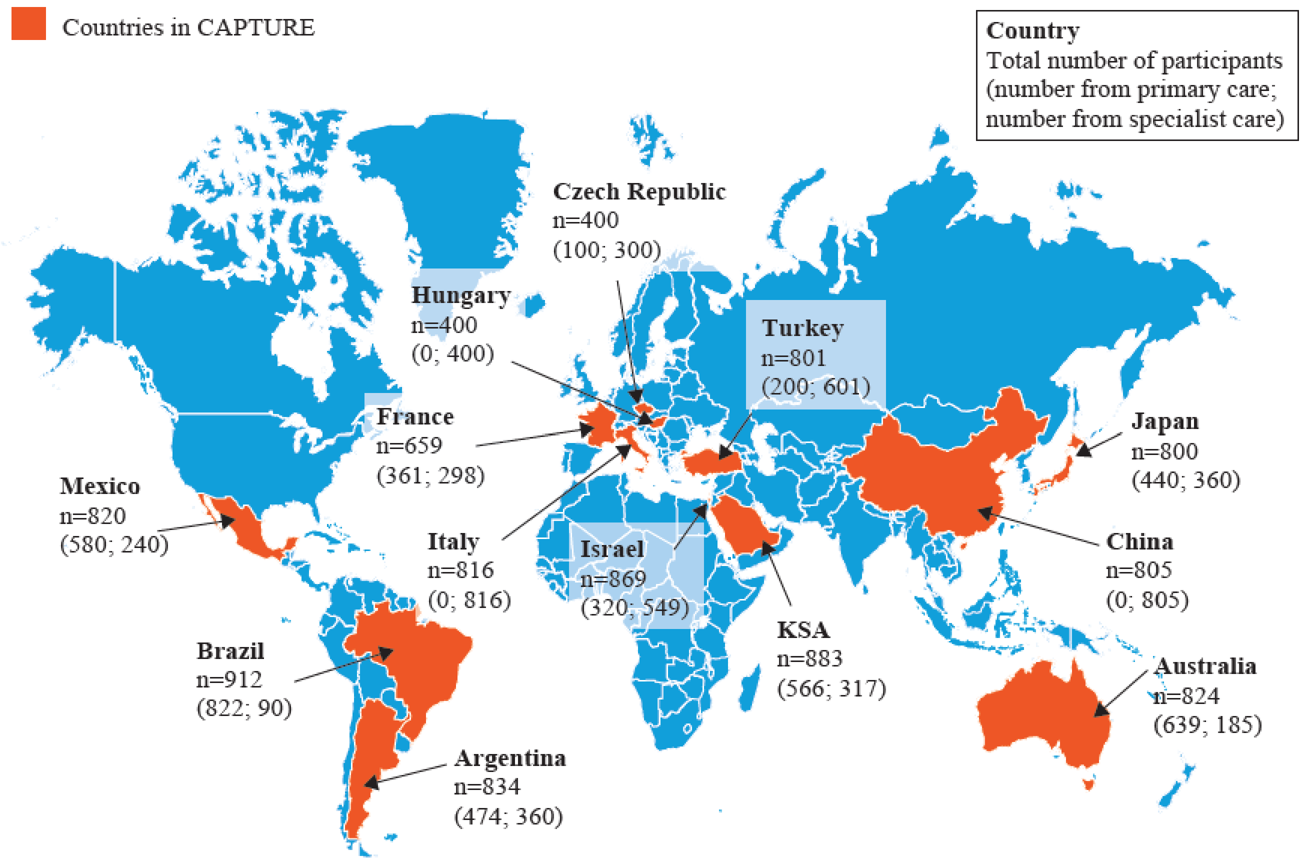

KSA Kingdom of Saudi Arabia

Figure S3. Weighted prevalence of heart failure in people with type 2 diabetes across the 13 countries (by country and overall)

Region	Country (number of participants; estimated diabetes population size)	n	Prevalence estimate [95% CI], %
Australasia	**Australia** (824; 1,133,000)	67	8.1 [6.3; 9.9]
East Asia	**China** (805; 114,394,800)	2	0.2 [0.0; 0.9]
	**Japan** (800; 7,234,200)	19	2.4 [1.4; 3.4]
Europe	**Czech Republic** (400; 767,800)	16	4.0 [2.1; 5.9]
	**France** (659; 3,276,400)	28	4.6 [2.9; 6.2]
	**Hungary** (400; 706,800)	22	5.5 [3.5; 8.2]
	**Italy** (816; 3,402,300)	34	4.2 [2.9; 5.8]
Latin America	**Argentina** (834; 1,757,500)	47	5.7 [4.1; 7.2]
	**Brazil** (912; 12,465,800)	113	12.4 [10.4; 14.4]
	**Mexico** (820; 12,030,100)	68	8.4 [6.8; 10.0]
Middle East	**Israel** (869; 415,800)	112	14.5 [11.6; 17.4]
	**KSA** (883; 3,852,000)	8	0.9 [0.3; 1.6]
	**Turkey** (801; 6,694,400)	43	5.4 [3.8; 6.9]
Overall	**Overall** (9823; 168,130,900)	579	2.4 [2.1; 2.7]

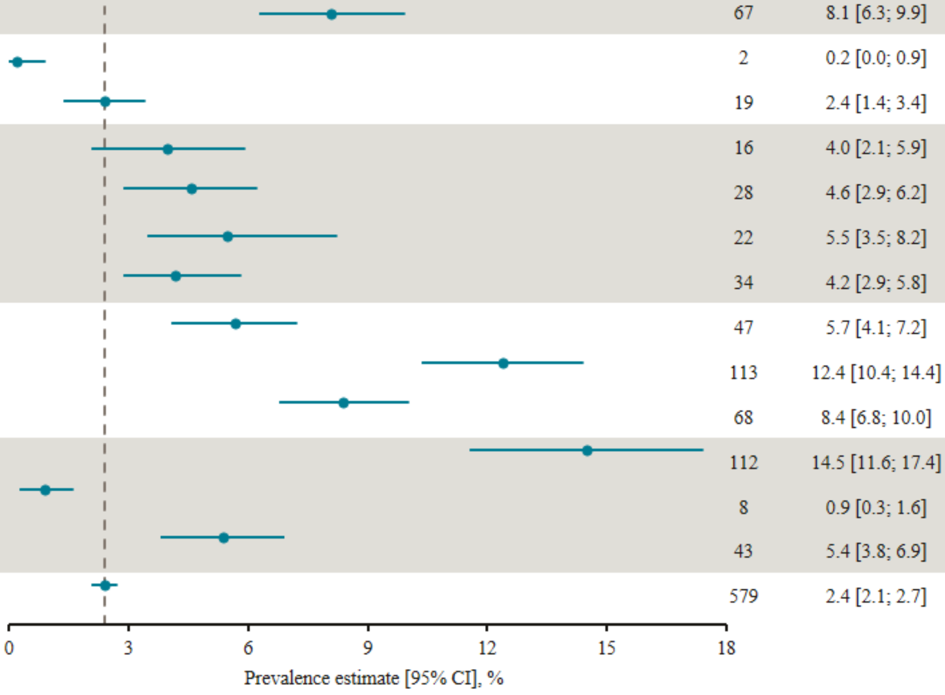

The overall prevalence estimate (across the 13 countries) was calculated as a weighted estimate to account for the size of the diabetes population of each country [ref 1] and is represented by the grey dotted line. Both the overall and country-level prevalence estimates were weighted by the sampling of participants by healthcare setting, if it was different from as planned. n numbers are the crude number of participants with heart failure (i.e. they were not weighted)

CI confidence interval, *KSA* Kingdom of Saudi Arabia, *n* number of participants with heart failure

Figure S4. Number of vascular areas affected among participants with CVD, stratified by gender

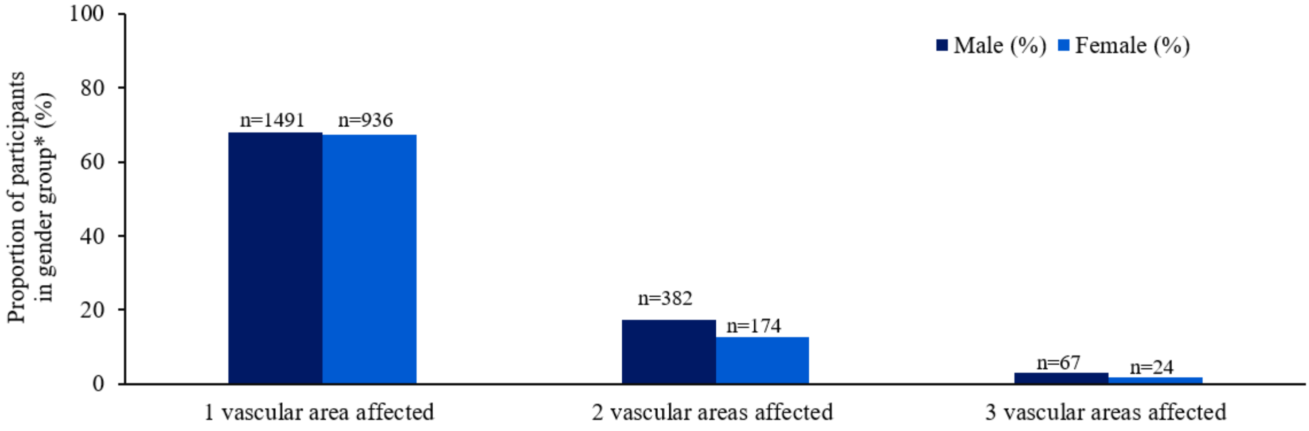

Vascular areas were defined as coronary, cerebrovascular or peripheral. *Information on vascular beds was available for 1134 females and 1940 males

CVD cardiovascular disease

Figure S5. Adjusted associations between country and CVD prevalence (prevalence odds ratios) – covariates identified via logistic regression

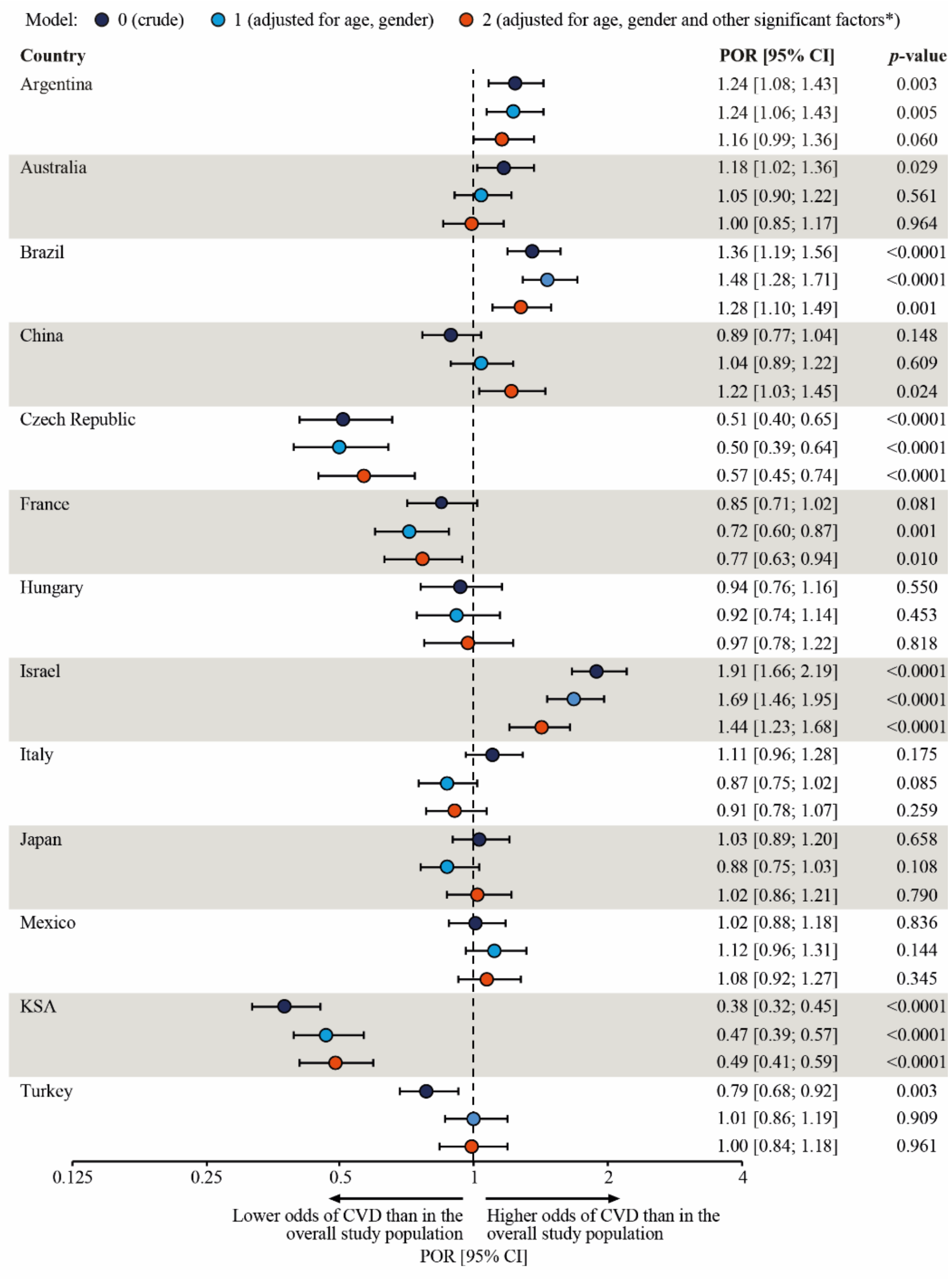

Analysis set (n = 9,713). Data are POR [95% CI] from logistic regression models with CVD (yes/no) as dependent variable and the following covariates: model 0, country; model 1, country, gender and age; *model 2, country, gender, age, diabetes duration, body mass index, HbA_{1c}, low-density lipoprotein cholesterol, high-density lipoprotein cholesterol, smoking status, hypertension,

nephropathy, neuropathy, and retinopathy. Missing data were imputed by fully conditional specification. The x-axis uses a log (base 2) scale

CI confidence interval, *CVD* cardiovascular disease, HbA_{1c} glycated hemoglobin, *KSA* Kingdom of Saudi Arabia, *POR* prevalence odds ratio

Figure S6. Sensitivity analysis. Adjusted associations between country and CVD prevalence (prevalence odds ratios) – covariates identified via logistic regression without imputation of missing data

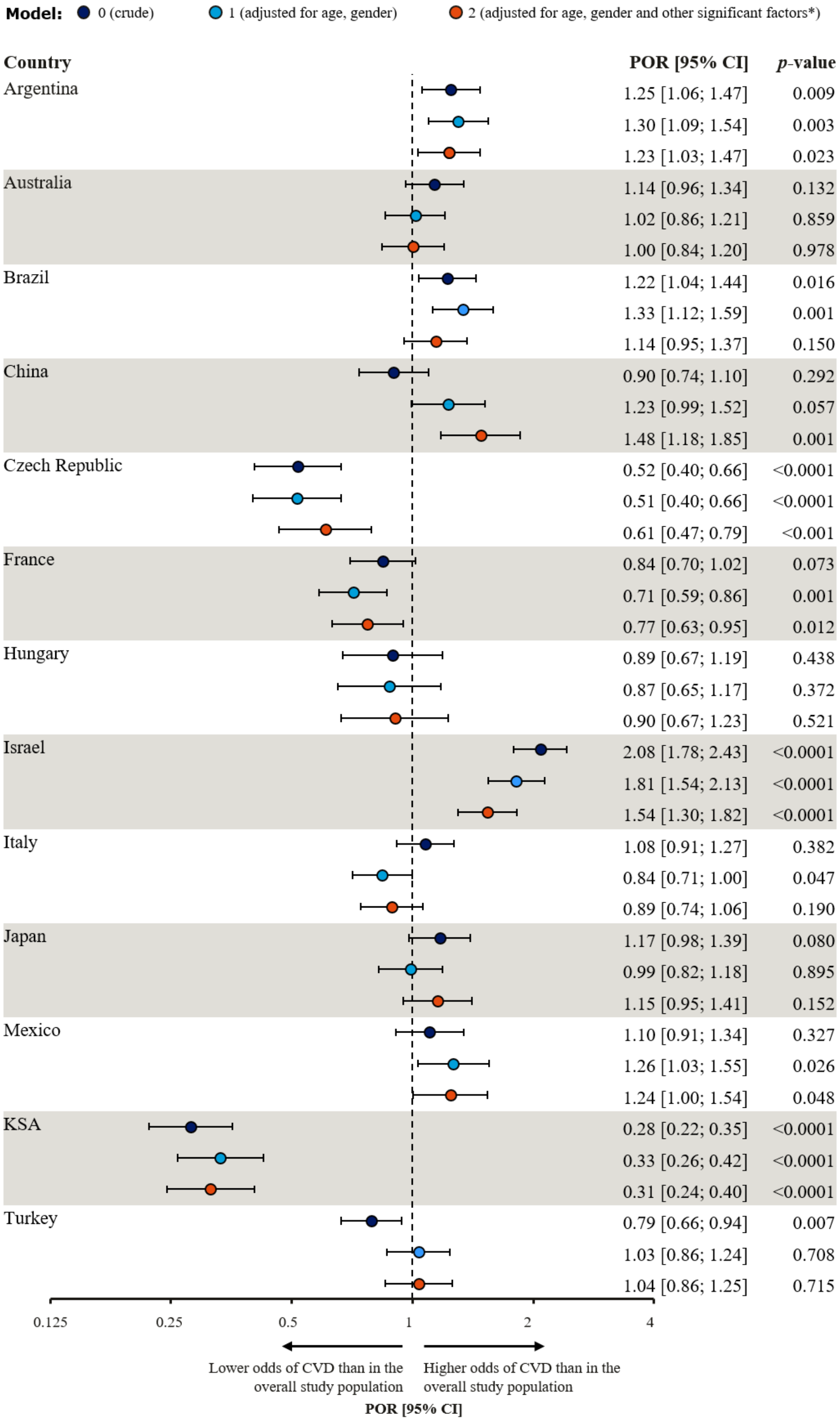

Analysis set (n = 7,163) with complete covariate information. Data are POR [95% CI] from logistic regression models with CVD (yes/no) as dependent variable and the following covariates: model 0: country; model 1: country, gender, and age; *model 2: country, gender, age, diabetes duration, body mass index, HbA_{1c}, low-density lipoprotein cholesterol, high-density lipoprotein cholesterol, smoking status, hypertension, nephropathy, neuropathy, and retinopathy. Missing data were not imputed. The x-axis uses a log (base 2) scale

CI confidence interval, *CVD* cardiovascular disease, *HbA_{1c}* glycated hemoglobin, *KSA* Kingdom of Saudi Arabia, *POR* prevalence odds ratio

Figure S7. Sensitivity analyses. Adjusted associations between country and CVD prevalence (prevalence odds ratios) – covariates identified via logistic regression (including eGFR)

A) With imputation of missing data

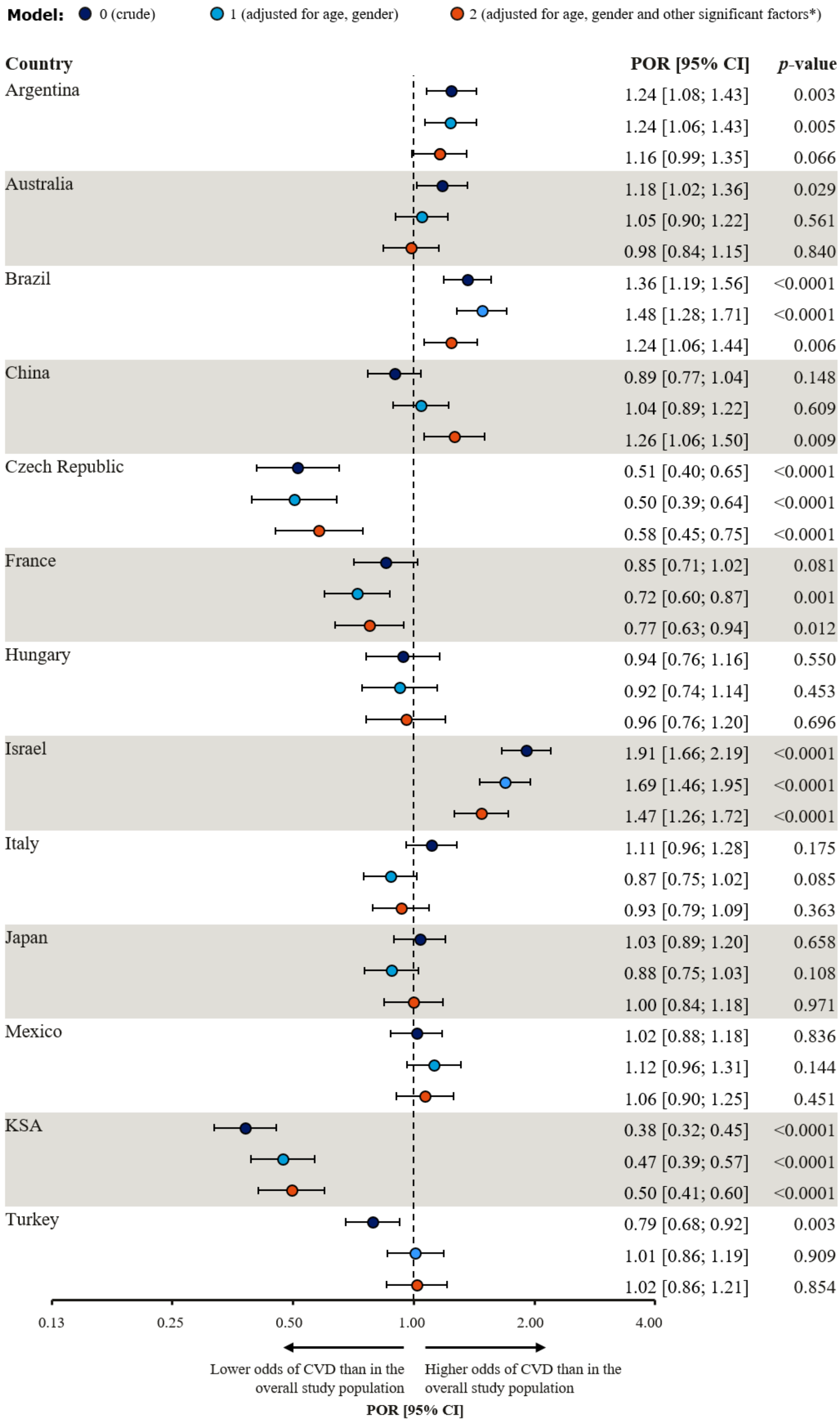

B) Without imputation of missing data

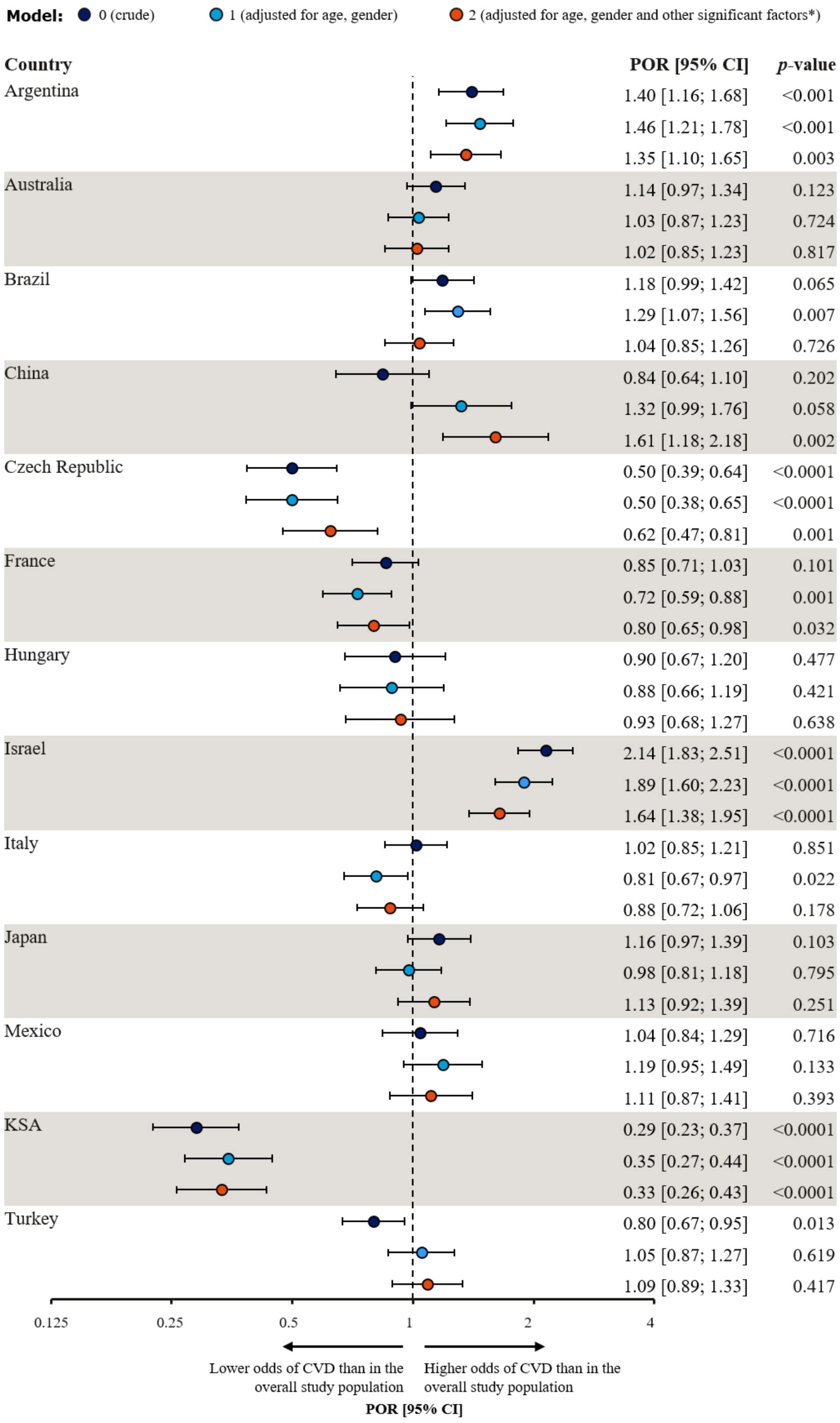

Analysis set (A: n = 9,713; B: n = 6,369 with complete covariate information). Data are POR [95% CI] from logistic regression models with CVD (yes/no) as dependent variable and the following

covariates: model 0: country; model 1: country, gender, and age; *model 2: country, gender, age, diabetes duration, body mass index, HbA_{1c}, eGFR, low-density lipoprotein cholesterol, high-density lipoprotein cholesterol, smoking status, hypertension, nephropathy, neuropathy, and retinopathy. In A: missing data were imputed by fully conditional specification; in B: missing data were not imputed. The x-axis uses a log (base 2) scale

CI confidence interval, *CVD* cardiovascular disease, *eGFR* estimated glomerular filtration rate, HbA_{1c} glycated hemoglobin, *KSA* Kingdom of Saudi Arabia, *POR* prevalence odds ratio

Additional reference

1. International Diabetes Federation. IDF Diabetes Atlas, 8th edn. Brussels, Belgium: International Diabetes Federation; 2017.

List of CAPTURE study investigators/collaborators

Argentina: Alejandra Oviedo, Martha Calveyra, Veronica Geronazzo, Natalia Nardelli, Paola Palermo, Cenudiab (Medical Center Diabetes And Nutrition); Jorge Waitman, Silvana Ernestina Solis, Jose Maria Pozzi, Centro Diabetologico Córdoba Dr. Waitman; Silvia Inés Orio, Blanca Acela Ozuna, Investigaciones Médicas IMOBA S.R.L.; Miguel Bustamante Labarta, Karina Gallardo, Maria Jose Pelagagge, Centro Medico Lebensohn; Claudia Baccaro, Veronica Lacoste, Mariano Chahin, Centro de Investigación Medico Lanus-CIMEL; Virginia Visco, Alejandra Camino, Gustavo Costa, Claudia Crespo, Dim Clinica Privada; Virginia Sernia, Instituto de Investigaciones Clínicas Mar del Plata; Pedro Rosario Fabián Calella, CIPADI Centro Integral de Prevención y Atención en Diabetes; Adriana Villarino, Susana Bertola, Ángeles Capizzelli, Carolina Faria, Instituto Medico DAMIC S.R.L.; Gloria Vines, Agustín Ramón, Hospital Privado de Comunidad; Laura Maffei, Emilio Alaguibe, Maria Coronel, Pedro Federico Cruz, Santiago Frid, Graciela Perez Prados, Claudia Fernandez Imaz, Investigación Clínica Aplicada; Guillermo Dieuzeide, Carolina del Carmen Gonzalez, Centro De Atención Integral En Diabetes; Lucrecia Nardone, Laura Nardone, Fundacion CEMAIC; Luis De Loredo, Silvina Daluz, Milena Suppo, Maria Teresa Cismondi, Hospital Privado Centro Médico de Córdoba S.A.; Sonia Hermida, Graciela Gili, Evangelina Buso, Evangelina Veronica, Ciad-Consutorio Integral De Atención Al Diabético; Diego Aizenberg, Fiorella Argento, Leandro Ianni, Martin Suarez, Centro Medico Viamonte; Alberto Caccavo, Virginia Alarcia, Andrea Alebuena, Juan Benger, Maria Arzadun, Clínica Coronel Suarez S.A.; Claudia Lamela, Patricia Castaño, Instituto Medico Especializado (IME).

Australia: Mark Kotowicz, Shannon McCarthy, University Hospital Geelong Barwon Health, Clinical Trials; Alexander Neville, Ferdinandus De Looze, AusTrials; Robert Moses, Jane Zhang, Alexia Pape, Zoran Apostoloski, Illawarra Diabetes Service Clinical Trial And Research Unit; Michael d'Emden, Royal Brisbane Women's Hospital; Ktut Mark Arya, Jason Doong Hing Kiang, Australian Clinical Research Network (ACRN); Peter Colman, Spiros Fourlanos, Royal Melbourne Hospital; Naveen Thalluri, Laurence Kemp, Alexander Neville, Suzanne Niven, Kenneth Purdie, Chuan Phing Tang, Tullio Testa, Sabitha Yakkala, Victoria Point Medical and Dental; Lakshman Prasad, Daniela Radulescu, Mihail Radulescu, Coffs GP Superclinic; Hamid Assaf, Hiwa Sabir, Stephen Williams, Moonee Ponds Medical Centre; Mazharul Islam, Mario Marazita, High Street Medical & Dental Centre Preston; Clive Tucker, Temba Dhobha, Alexander Neville, Riverlink Medical and Dental; Kamal Deep Kaur, Qui-man Zhao, Medical & Dental Centre; John Wong, Usman Rana, Shahed Alam, Seymour Street Medical & Dental; Nathan Zhou, Hussain Ahmed, Sharmin Akhter, Essop Ebrahim, Nazish Fatima, Zaheed Hossain, Bashir Raji, Subash Mawji, Campbelltown Medical & Dental Centre; Timothy Davis, Fremantle Hospital and Health Service; Antony Shea, Ryan

Dougherty, Rina Kennedy, Kelvin Matingo, Renu Rayani, Edgardo Salamat, Morayfield Rd Medical and Dental; Martin Jodlowski-Tan, Alexander Neville, Pacific Medical Centre Blacktown; Janine Teasdale, Warringah Medical and Dental Centre.

Brazil: Marcelo Hissa, Miguel Nasser Hissa, Centro De Pesquisas Em Diabetes E Doencas Endocrino Metabolicas Ltda.; Dalton Precoma, Antonio Acosta Pazzini, Janaina Bernardi Barea, German Gonzales, Camila Richter, Sociedade Hospitalar Angelina Caron; Maria Helena Vidotti, Fabio Rossi Dos Santos, Loema Medicina e Bem Estar - Centro Especializado em Cardiologia Ltda; Livia Figueira Avezum Oliveira, Jose De Oliveira Ferreira, Paulo Maia Vilela, Elisabete Mantovany Rodrigues de Resende, Hospital de Clinicas da Universidade Federal do Triangulo Mineiro; Flavia Coimbra Pontes Maia, Angelica Maria Paiva Tiburcio, Santa Casa De Misericordia de Belo Horizonte; Mirian Hideco Takahashi Albuquerque, Emilio Carlos Zequim, Wilson Eik Filho, Clinica e Pesquisa em Endocrinologia de Maringa SS; Gracielly de Souza Pantano, Mauro Esteves Hernandes, Santa Casa de Misericordia de Votuporanga; Freddy Eliaschewitz, Renata de Carvalho Camara Bona, Fabiana De Almeida Palhares, Guilherme De Lima Visconti, Vanessa De Oliveira Magalhaes, Denise Franco, Denise Ludovico da Costa de Castro, Flora Marcolino, Katia Nunes de Siqueira, CPClin- Centro de Pesquisas Clinicas Ltda./Clinica Dr. Freddy Goldberg Eliaschewitz; Estevao Lanna Figueiredo, Gualter Cancado, Gustavo Micena Araujo, Fernando Neuenschwander, Nucleo de Pesquisa Clinica do Hospital Vera Cruz; Daniela Espindola Antunes, Guillerme Burges, Hospital das Clinicas da Universidade Federal de Minas Gerais (HCUFMG); Tania Maria Bulcao Lousada Ferraz, Instituto de Estudos e Pesquisas Clinicas do Ceara (IEP); Miguel Riella, Ana Campanholo, Gina Morgano, Instituto Pro-Renal Brasil; Hugo Roberto Kurtz Lisboa, Luciano Backes, Milena Rodrigues, Cintia Tavares, Glaucia Tres, Hospital Sao Vicente de Paulo - Bioserv SMO; Lenara Golbert, Milton Moyses Golbert, Irmandade Sta Casa Misericordia Poa - Centro Medico; Wladmir Saporito, Ana Luiza Campanholo, Gina Morgro Gordon, Luciana de Oliveira, Instituto de Molestias Cardiovasculares Tatui; Teresa Piscitello Bonansea, Maria Nunes, Marta Eliane de Carvalho Bastos, Luana Casari Da Silva Lima, Andrea Barranjard Vannucci Lomonte, CEPIC - Centro Paulista de Investigacao Clinica e Servicos Medicos Ltda.; Antonio Da Silva, Adolf Franco, Clinilive (Clinica do idoso e pesquisa clínica); Marcio Krakauer, Lia Lima de Araujo Cals, Leticia Figueiredo Vilela, Hospital e Maternidade Dr. Christovao da Gama; Vianna Andre, Camile Cruzeta Vasconcellos, Marcela Da Silva, Claudio De Lacerda, Josiane Cristine Detsch, Caroline Giocondo, Juliana Lehmkuhl, Andressa Leitao, Emerson Marino, Kleber Marques, Luciana Pechmann, Michelle Polesel, Claudia Sanches Rocha, Felipe Teixeira, Centro de Diabetes Curitiba; Luis Canani, Adriana Valenti, Carla Camacho Sartori, Nucleo de Pesquisa Clinica do RS; Sergio Cunha Vencio, Marianne Cruz, Instituto de Ciencias Farmaceuticas – ICF.

China: Tian-Pei Hong, Chen Wang, Guoqiang Liu, Jingjing Zhang, Jin Yang, Junling Liu, Linxi Zhang, Qing Tian, Ran Lu, Weiguan Du, Wenfang Hou, Wenhua Xiao, Xiaoxia Wang, Yanrong Wang, Ye Liu, Chao Xie, Peking University Third Hospital; Wei Tang, Lingli Dai, Cuining Hu, Qinglin Lour, Kunlin Wang, Dan Wu, Ting Xu, Yun Yu, Shan Shan, Cuinin Hu, Jiangsu Province Official Hospital; Li Li, Yuchen Tang, Lingyun Wu, Miao Xu, Ningbo First Hospital; Qin Wan, Xue Bai, Pan Chen, Wei Huang, Hua Li, Hongyan Ma, Pijun Yan, Xiaojie Wang, Ying Miao, Jun Yang, Ling Xu, Chenlin Gao, Southwest Medical University Affiliated Hospital; Lin Qi, Dejun Hao, Dongya Zhang, Pijun Yan, Jianhua Suo, Jie Zou, Wenhui Zhang, Xiangdong Wang, Yongli Yu, Youjun Zhang, Guijing Qu, Beijing Yanhua Hospital; Zongxun Yan, Yihua Peng, Na Zhang, Lin Liu, Qiaoyan Luo, Xingmin Zhang, Yuhua Chen, Affiliated Hospital of North Sichuan Medical College; Jiangong Ren, Yanling Feng, Dan Ge, Yuanhua Hou, Xuejian Hu, Gengxue Jin, Yifan Liu, Furong Ma, Qi Ma, Wenting Tie, Yanmei Wang, Ruihong Yue, Ming Zhang, Tao Zhang, Ruihong Guo, Hui Chen, The Second Hospital of Lanzhou University; Jianhua Ye, Chenghong Ma, Chou Liao, Li Hu, The First Affilliated Hospital with Guangdong Pharmaceutical University.

Czech Republic: Tomas Brychta, Agentura Science Pro; Jana Horackova, Jozefina Stefankova, StefaMed; Pavlina Kyselova, DIEKO; Vitezslav Mejzlik, MUDr; Michal Brada, Diabetologicka ambulance Breclav; Dita Pospisilova, DIAMEDICA; Jana Bomberova, MUDr; Sarka Kopecka, DIARESEARCH; Dagmar Kopejtkova, Dia poradna Chomutov; Katerina Urbancova, Diabetologicka interni ambulance; Igor Karen, Ordinace praktickeho lekare; Anna Kortova, AK Medipraktik; Jana Vojtiskova, Hana Benesova, Lekarska ordinace Vysehrad; Hugo Pribyl, MUDr, Ph.D; Dana Moravcikova, MUDr.

France: Patrice Darmon, CHU La Conception - Endocrinology Service de Nutrition, Maladies Métaboliques, Endocrinologie; Marc Flosi, Cabinet Medical Flosi; Lionel Specht, Cabinet Medical Marlier; Pierre Serusclat, Patricia Carre, Groupe Hospitalier les portes du sud – Endocrinology; Laurent Meyer, Centre d'Endocrinologie-Diabete-Maladies Metaboliques; Dominique Delsart, Cabinet Medical Delsart; Yanis Peyrol, Cabinet Medical Peyrol; Pascal Mabire, Cabinet Medical Mabire; Said Bekka; Practice Said Bekka; Sylvaine Clavel, Fondation Hotel Dieu; Bertrand Lammens, Cabinet Medical Lammens; Antoine Franceschini, Cabinet Medical Franceschini; Frédéric Gehin, Cabinet Medical Gehin; André Sebbah, Cabinet MÃ©dicale; Chantal Simonet-Thomas, Private Praxis Dr. Chantal Simonet-Thomas; Myriam Deschamps-Ben Ayed, Practice Dr. Deschamps-Ben Ayed; Sylvie Marsot, Private Consultation Dr. Marsot; Lucien Marchand, Hôpital privé St Joseph St Luc – Endocrinology.

Hungary: Csaba Lengyel, Szabolcs Nyiraty, Tamás Várkonyi, Bettina Tóth, Szegedi Tudomanyegyetem Altalanos; Tamas Oroszlan, Zoltan Bujtor, Zoltan Bagosi; Zala Megyei Szent Rafael Korhaz, Zala Megyei Szent Rafael Korhaz II; Gabor Simonyi, Roland Gasparics, Szent Imre Egyetemi Oktatokorhaz; Zsolt Hermanyi, Péter Torzsa, Kris-Med Orvosi és Tanácsadó Bt. – Endocrinology, Bajcsy-Zsilinszky Korhaz es Rendelointezet III; Viktor Vass, Andrea Filo, XVI. Kerület Kertvárosi Egészségügyi Szolgálata – Diabetológia, Synexus Magyarorszag Kft. Hungary Clinical Research Site; Piroska Turbucz, Sándor Szépkúti, Pest Megyei Flor Ferenc Korhaz; Denes Pall, Tamás Köbling, Regina Esze, University of Debrecen, Debreceni Egyetem Klinikai Kozpont; Enikő Dezső, László Deak, Somogy Megyei Kaposi Mor Oktato Korhaz; Zoltan Marton, Flóra Bettina Petró, Toldy Ferenc Korhaz es Rendelointezet; Marietta Baranyai, Eva Viragh, Markusovszky Egyetemi Oktatókórház.

Israel: Adiv Goldhaber, Amdur Lubov, Kaminsky Ruslana, Ariella Seidman, Shuali Community Clinic - Clalit Health Services; Daniel Tzalichin, Yud Alef Cummunity Clinic - Clalit Health Services; Muhammad Sabbah, Michal Gershinsky, BaruchYitzhak, Diabetes Unit, Lin Clinic Carmel Medical Center; Mahmud Darawsha, Khaled Badarnih, Saed Darawshi, Sachnin Clinic - Clalit Health Services; Adnan Zaina, Endocrinology & Metabolism Department - Zvulun Community Medical Center - Clalit Health Services; Jarir Khury, Nazareth Towers Clinic - Clalit Health Services; Anat Tsur, Hashem Bseiso, Susan Chayen, Odile Cohen-Ouaknine, Asher Prosper Corcos, Meir Frankel, Arin Haj Yahia, Ofa Makhoul, Muriel Metzger, Victoria Terletsky, Hamoshava Endocrinology and Diabetes Clinic - Clalit Health Services; Rosane Abramof Ness, Lidia Diamant, Yoav Eizenberg, Dana Herzberg, Moshe Karp, Sapir Mzia, Migdal Hamea Medical Center - Clalit Health Services; Gallina Abramov, Hashalom Clinic - Clalit Health Services; Julio Wainstein, Lea Chananashvili, Itzhak Bar Or, Yosefa Bar Dayan, Diabetes Unit, Wolfson Medical Center; Faiad Adawi, Endocrinology and Diabetes Center, Ziv Medical Center; Ilan Shimon, Chagit Adler Cohen, Joelle Attal-Singer, Hadar Duskin-Bitan, Alexander Gorshtein, Dania Hirsch Kolerman, Hiba Maski-Iraqi, Eyal Robenshotok, IIana Slutsky, Gloria Tsvetov, Institute of Endocrinology, Diabetes & Metabolism, Rabin Medical Center, Beilinson Campus; Amir Bashkin, Natalia Kaykov, Marina Nodelman, Diabetes and Endocrinology Unit, Galilee Medical Center; Ofri Mosenzon, Marina Potekhin, Matan Fischer, Diabetes Unit, Hadassah Medical Center; Ilana Shraga-Slutzky, Ylena Zbolonov, Bat Yamon Clinic - Clalit Health Services; Alon Grossman, Tomer Arad, Ben Cohen, Navad Mizrahi, Felix Mor, Internal Medicine B, Rabin Medical Center, Beilinson Campus; Eliezer Klainman, Gefen Institute, Rafael Medical Center.

Italy: Giuseppina Russo, Roberta Serra, Policlinico Messina; Marco Mirani, Istituto Clinico Humanitas; Gabriella Giuseppina Maria Garrapa, Azienda Ospedaliera Ospedali Riuniti; Maria Gisella Cavallo, Laura Bertoccini, Flavia Cimini, Policlinico Umberto I; Marcello Monesi, Arcispedale S.Anna, Azienda Ospedaliero Universitaria di Ferrara; Franco Arturi, Azienda Ospedialero Universitaria Mater Domini; Salvatore Oleandri, Ospedale Civile SS Annunziata; Giuseppe Memoli, Centro Antidiabetico San Luca; Luciano Zenari, Ospedale Sacro Cuore Don Calabria; Gerardo Corigliano, Marco Corigliano, Centro Associazione Nazionale Italiana Atleti Diabetici; Francesco Manetti, Ospedale Santa Maria Annunziata; Rinaldo Guglielmi, Ospedale Regina Apostolorum; Vincenzo Provenzano, Fleres Mattia, ospedale ASP-6; Olga Disoteo, Ospedale Niguarda Ca' Granda; Francesca Pellicano, Diabetologia Ravenna AUSL della Romagna; Antonio Caretto, Ospedale Perrino; Massimo Bracaccia, Ospedale Santa Maria Della Stella; Edoardo Mannucci, Azienda Ospedaliero Universitaria Careggi.

Japan: Yukiko Onishi, Kazuyuki Takahashi, Asuka Shinmel, The Institute for Adult Diseases Asahi Life Foundation; Shinichiro Shirabe, H. E. C Science Clinic; Hiroaki Seino, Seino Internal Medicine Clinic; Hideto Ishii, Hiroyuki Asano, Asano Clinic; Yoko Abe, Nobuyuki Abe, Katsushige Abe, Rie Abe, Abe Clinic; Kazushi Amano, Heiwadai Hospital; Hideaki Jinnouchi, Jinnouchi Hospital; Yasuhiro Ono, Iryo Houjin Syadan Kouhoukai Takagi Hospital; Shuichi Fukuda, Wakakusa Clinic; Kiyokazu Matoba, Matoba Diabetes Clinic; Yasuo Sato, Sato Clinic; Osamu Tomonaga, Medical Corporation Group Lifestyle Tomonaga Clinic; Hidenori Ishida, Akaicho Clinic; Hiroshi Ohashi, Oyama East Clinic; Shin-Ichiro Satoh, Miyanosawa Clinic of Internal Medicine and Cardiology; Takashi Sasaki, Katsuhisa Ishii, Keiko Sakai, Sasaki Internal Medicine; Madoka Fujimoto, Koshigaya Laketown Clinic; Nobuyuki Sato, Toshihisa Matsuda, Sato Internal Medicine Clinic; Hiroko Ohishi, Hamamatsu Rosai Hospital; Shinya Nakamoto, Nakamoto Internal Medicine Clinic.

Mexico: Enrique Cuitláhuac Morales Villegas, Alejandra Aburto Ituarte, Gualberto Moreno Virgen, Rubria Ramirez Baez, Jorge Vital, Centro de Investigación Cardiometabólica de Aguascalientes SA de CV; Ignacio Rodriguez Briones, Raul Estrada Garcia, Jose Alfredo Haro Ortiz, Cardioarritmias e Investigación S.C.; Sigfrido Miracle Lopez, Marco Antonio Segovia Rivas, Sigfrido Miracle Lopez Consultorio Médico 345 Del Hospital Ángeles Lomas; Diego Espinoza Peralta, Ana Gisela Noriega Muñoz, Investigación Médica Sonora S.C.; Guillermo Gonzalez-Gálvez, Martha Lopez Velasco, Iliana Pazos Velasco, Gabriela Ramos Aldrete, Blanca Sanchez Michel, Instituto Jalisciense de Investigación en Diabetes y Obesidad S.C.; Ernesto German Cardona Muñoz, Maria Ramos Zavala, Icle S.C.; Azael Gilberto Vidal Garcia, Adriana Balderrama Soto, Brenda Garcia Gil, Consultorio Médico Dra. Adriana Balderrama Soto; Natalia De la Garza Hernandez, Centro Medico Integral;

Carlos Alberto Domínguez-Reyes, Julieta Gonzalez Palacios, Centenario Hospital Miguel Hidalgo; Juan Carlos Vázquez Ávila, Angel Jair Marquez, Consultorio Privado del Dr. Juan Carlos Vázquez Ávila; Guillermo Fanghänel Salmon, Marxivonne Diaz Espinoza, Leticia Sanchez Reyes, Clínica Integral del Paciente Diabético y Obeso; Sara Pascoe Gonzalez, Sandra Ofelia Hernandez, Instituto de Terapéutica Experimental y Clínica (INTEC) Centro Universitario de Ciencias de la Salud; Carlos Jerjes Sanchez Díaz, Héctor Rodriguez Flores, Alicia Ramírez, Susana Ramírez Rivera, Unidad de Investigación Clínica en Medicina, S.C.; Juan Antonio Peralta Calcaneo, Valentín Pedraza, Hospital General de Mexico (HGM); Juan Rosas Guzmán, Juan Rosas Saucedo, Instituto de Diabetes, A.C.; Jose Luis Arenas León, Yunnuen Martínez, Jose de Jesús Rivera, Centro de Atención e Investigación Cardiovascular del Potosí S.C.; Israel Olvera Álvarez, Anaid Galindo Pérez, Norma Martínez Trejo, Clínicos Asociados BOCM, S.C.; Leobardo Sauque Reyna, Aurelia Baez Campos, Jesús Moran Farías, Instituto de Diabetes, Obesidad y Nutrición S.C.; Fernando Flores-Lozano, Ángela Cabeza Gomez, Celia Anaya Preciado, Ana Maria Barragan Vigil, Elena Calardo Zakari, Hospital Dr. Ángel Leaño; Rosa Isela Luna Ceballos, Josue Grisffed Montiel Vázquez, Sociedad de Metabolismo y Corazón S.C.; Susano Lara Vaca, Talitha Gumi Vallejo, Consultorio Privado del Dr. Susano Lara Vaca; Raymundo Garcia Reza, Humberto Javier Rivera, UBAM Unidad Biomédica Avanzada Monterrey; Rafael Margarito Violante Ortiz, Denisse Castillo Reveles, Sarah Chong Garcia Torres, Jordi Gutierrez Sanchez, Jorge Luis Lopez Lu, Daniela Mendez, Centro de Estudios de Investigación Metabólicos y Cardiovasculares S.C.; Gerardo Juan Gonzalez Girón, Adrián Garcia Carreón, Antonia Jiménez Cienfuegos, Miriam Valdez Beltrán, Centro de Investigación Clínica del Pacífico, S.A. de C.V.; Carlos Hernandez Herrera, José Hernandez Carrizales, Instituto Cardiovascular de Monclova, S de R.L de C.V.; Gabriel Arturo Ramos Lopez, Liliana Estefania Ramos Villalobos, Private office Dr. Gabriel Arturo Ramos Lopez; Manuel Duarte Vega, Jesús Santiago Delgadillo Centeno, Private office Dr. Manuel Duarte Vega; Silvia Alejandrina Jiménez Ramos, Diana Larios Mora, Alan Fernando Zargen Ramirez, Centro de Investigación Clínica Endocrinológica de Jalisco S.C.; Manuel Odin de Los Rios, Laura Serrano Quintana, Juan Rodriguez, Raul Rojo Medina, Centro para el Desarrollo de la Medicina y de Asistencia Medica Especializada S.C.; Nacu Aureo Caracas Portilla, Marisela Jiménez Sanchez, Hospital Angeles Lindavista Endocrinología y Nutriología; Marco Antonio Alcocer Gamba, Claudia Patricia Aguado Herrera, Centro de Estudios Clínicos de Querétaro S.C.; Jorge Chávez Páez, Nestor Alonso López Rubio, Instituto de Diabetes y Riesgo Cardiovascular S.A de C.V.

Saudi Arabia: Saud Sefari, Alhada Armed Forces Hospital; Turki Almigbal, Mohamed Batais, Abdullah Alguwaihes, King Khaled University Hospital, King Saud University; Nader Al Balkhi, Specialized Medical Centre Hospital; Amany Al Hozali, King Abdulaziz University Hospital; Mohamed Hassan Hatahet, King Abdulaziz Hospital for National Guard; Turki AlMogbel, Diabetes and Endocrinology Center-Buraidah; Abdullah Alguwaihes, Dallah Hospital.

Turkey: Basak Oezgen Saydam, Suleyman Cem Adiyaman, Riza Simsek, Dokuz Eylul Universitesi Tip Fakultesi Ic Hastaliklari Anabilim Dali Endokrinoloji Bilim Dali Inciralti; Alper Sonmez, Health Sciences Gulhane School of Medicine; Fahri Bayram, Erciyes University; Mehmet Akman, Marmara Universitesi Hospital; Sari Ramazan, Akdeniz Universitesi Tip Fakultesi Dumlupinar Bulvari Kampus, Endokrinoloji ve Metabolizma Hastaliklari Bilim Dali Kampus; Sargin Mehmet, Istanbul Medeniyet University Faculty of Medicine; Secil Arica, Zekiye Pelin Yagan, Okmeydani Research and Education Hospital; Ozgur Demir, Ankara University Medical School; Dilek Toprak, Namık Kemal University Hospital; Altug Kut, Baskent University Ankara Hospital; Sibel Guldiken, Trakya Universitesi Tip Fakultesi Ic Hastaliklari Anabilim dali Endokrinoloji ve Metabolizma Hastaliklari Bilim Dali; Nur Kebapci, Eskisehir Osmangazi Universitesi Ic Hastaliklari Anabilim Dali Meselik Kampusu Eskisehir; Taner Bayraktaroglu, Zonguldak Bulent Ecev University, Obesity and Diabetes Application and Research Center, Kozlu Sahilyolu, Merkez Farabi Campus; Emir Donder, Hatice Rumeysa, Erhan Onalan, Firat Universitesi Tip Fakultesi Ic Hastaliklari Anabilim Dali Universite Mahallesi Yahya Kemal; Ibrahim Sahin; Turgut Ozal Tip Merkezi - Inonu Universitesi Fakultesi Hastanesi Endokrinoloji ve Metabolizma Hastaliklari Elazig Yolu.
